# Investigating the occurrence of autoimmune diseases among children and adolescents hospitalized for *Mycoplasma pneumoniae* infections

**DOI:** 10.3389/fimmu.2023.1165586

**Published:** 2023-12-05

**Authors:** Eun Kyo Ha, Ju Hee Kim, Hye Ryeong Cha, Bo Eun Han, Youn Ho Shin, Hey-Sung Baek, Sun Hee Choi, Man Yong Han

**Affiliations:** ^1^ Department of Pediatrics, Hallym University Kangnam Sacred Heart Hospital, Seoul, Republic of Korea; ^2^ Department of Pediatrics, Kyung Hee University Medical Center, Seoul, Republic of Korea; ^3^ Department of Computer Science and Engineering, Sungkyunkwan University, Suwon, Republic of Korea; ^4^ Department of Software, Sejong University, Seoul, Republic of Korea; ^5^ Department of Pediatrics, Yeouido St. Mary’s Hospital, The Catholic University of Korea, Seoul, Republic of Korea; ^6^ Department of Pediatrics, Kangdong Sacred Heart Hospital, Hallym University College of Medicine, Seoul, Republic of Korea; ^7^ Department of Pediatrics, Kyung Hee University Hospital at Gangdong, Seoul, Republic of Korea; ^8^ Department of Pediatrics, Bundang CHA Medical Center, CHA University School of Medicine, Seongnam, Republic of Korea

**Keywords:** autoimmune diseases, childhood, epidemiology, immune system, *Mycoplasma pneumoniae*, pneumonia

## Abstract

**Background:**

*Mycoplasma pneumoniae* infection is common in the general population and may be followed by immune dysfunction, but links with subsequent autoimmune disease remain inconclusive.

**Objective:**

To estimate the association of *M. pneumoniae* infection with the risk of subsequent autoimmune disease.

**Methods:**

This retrospective cohort study examined the medical records of South Korean children from 01/01/2002 to 31/12/2017. The exposed cohort was identified as patients hospitalized for *M. pneumoniae* infection. Each exposed patient was matched with unexposed controls based on birth year and sex at a 1:10 ratio using incidence density sampling calculations. The outcome was subsequent diagnosis of autoimmune disease, and hazard ratios (HRs) were estimated with control for confounders. Further estimation was performed using hospital-based databases which were converted to a common data model (CDM) to allow comparisons of the different databases.

**Results:**

The exposed cohort consisted of 49,937 children and the matched unexposed of 499,370 children. The median age at diagnosis of *M. pneumoniae* infection was 4 years (interquartile range, 2.5–6.5 years). During a mean follow-up time of 9.0 ± 3.8 years, the incidence rate of autoimmune diseases was 66.5 per 10,000 person-years (95% CI: 64.3–68.8) in the exposed cohort and 52.3 per 10,000 person-years (95% CI: 51.7–52.9) in the unexposed cohort, corresponding to an absolute rate of difference of 14.3 per 10,000 person-years (95% CI: 11.9–16.6). Children in the exposed cohort had an increased risk of autoimmune disease (HR: 1.26; 95% CI: 1.21–1.31), and this association was similar in the separate analysis of hospital databases (HR: 1.25; 95% CI 1.06–1.49).

**Conclusion:**

*M. pneumoniae* infection requiring hospitalization may be associated with an increase in subsequent diagnoses of autoimmune diseases.

## Introduction

*Mycoplasma pneumoniae* (*M. pneumoniae*) is one of the most frequent causes of respiratory diseases and is responsible for 10% to 40% of all cases of community-acquired pneumonia (CAP) in children. *M. pneumoniae*, a gram-negative bacterium lacking cell walls and characterized by its small size, depends on host cell association as an extracellular pathogen. It develops a specialized attachment organelle that potentially leads to the formation of capsular material outside the cell membrane. This bacterium is a significant respiratory pathogen in children (1). Although many infected individuals completely recover without complications, some experience sustained chronic diseases (2–4). More specifically, some children experience extrapulmonary manifestations that influence specific organs or whole-body systems after an infection. Some evidence suggests that a *M. pneumoniae* infection may lead to an increased development of autoimmune diseases (5).

There is increasing interest in the impact of *M. pneumonia* infection on autoimmune diseases (2, 6), and many case reports have examined this relationship (7, 8). However, there is no definitive evidence of a causal relationship because these studies had cross-sectional designs (9, 10), observational studies lacking appropriate controls (11), and examined small numbers of patients who were mostly from specialist clinics (12). There are also conflicting results from case–control studies regarding the relationship of *M. pneumoniae* infection with common childhood autoimmune diseases, such as Kawasaki disease and Guillain–Barre’s syndrome (13). We are unaware of any rigorous epidemiologic investigations that examined the effect of *M. pneumoniae* infection on subsequent autoimmune diseases.

The purpose of this study was to determine the association of *M. pneumoniae* infection with the risk of subsequent autoimmune diseases using a nationwide population-based database that has information on all medical diagnoses and use of healthcare resources, with control for confounding by comparison with hospital-based data.

## Methods

### Study design

This retrospective population-based cohort study enrolled all individuals born in South Korea between 2002 and 2005, as identified by the National Health Insurance Service (NHIS), with linked Statistics Korea census data. The NHIS provides healthcare services coverage for over 98% of the South Korean population and includes claims-based medical data. These data provided information on demographic characteristics, healthcare utilization (with diagnostic codes from the 10th version of the *International Classification of Diseases*, ICD-10), prescriptions, and relevant procedures. All guidelines for observational studies that use routinely collected health data were followed (**Supplementary Table 1**).

### Study population

**Figure 1:**
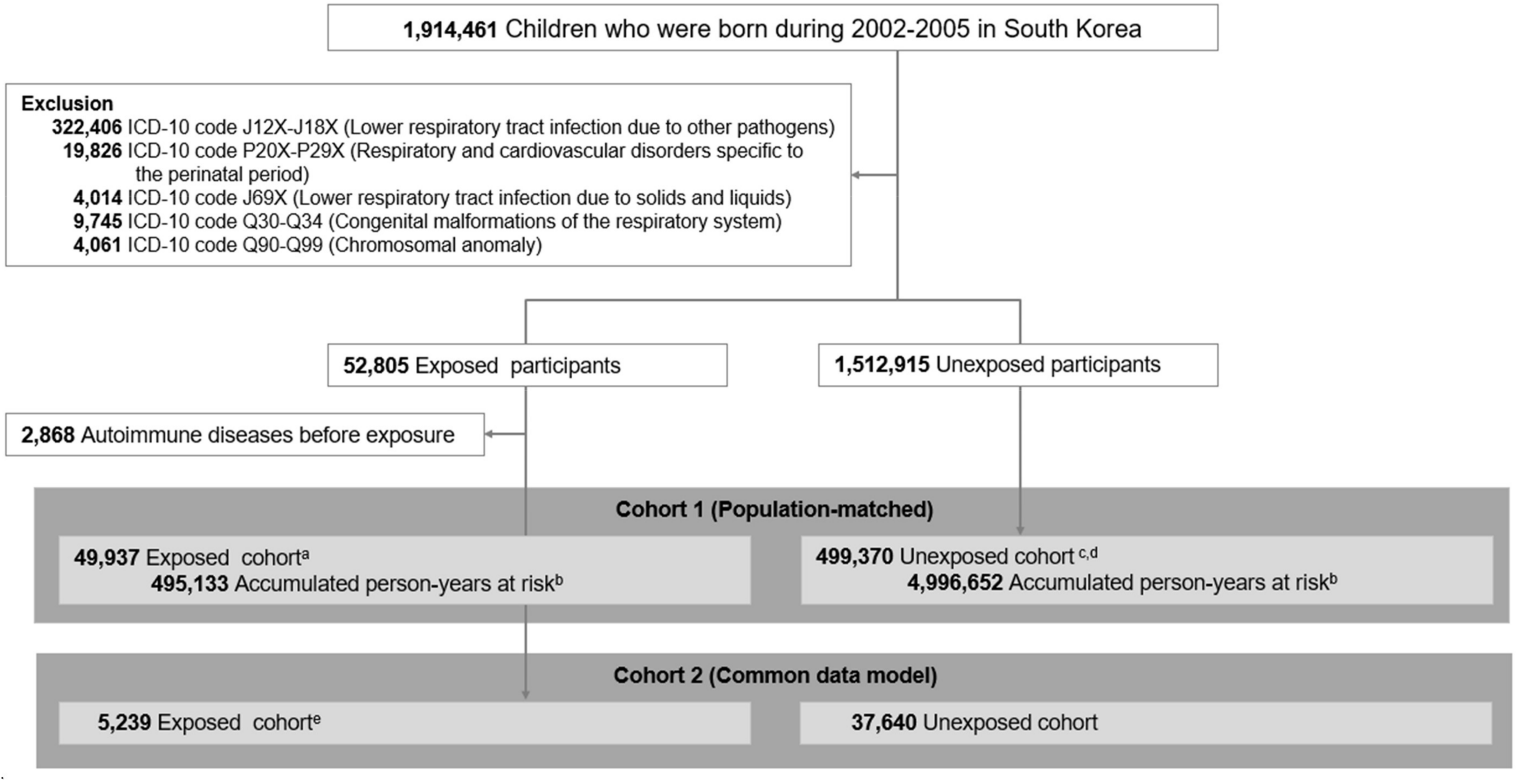
Study design. Individuals born in South Korea from 2002 to 2005 were identified from the database of the National Health Insurance Service (NHIS) and linked information on death from Statistics Korea. After application of exclusion criteria, there were 49,937 children in the exposed cohort and 1,277,613 matched children in the unexposed cohort. ^a^There were 265,430 born in 2002–2003, 233,940 born in 2004–2005. ^b^The first year of follow-up was excluded for calculation of accumulated person-years. ^c^Eligible unexposed individuals had no *M. pneumonia* infection and no autoimmune disease at the time of diagnosis of the matched index patient. Matching was performed using density sampling. There were 26,543 born in 2002–2003, and 23,394 born in 2004–2005. ^d^Among individuals in the unexposed cohort, 10,420 (2.1%) who received diagnoses of *M. pneumonia* infections during follow-up were reallocated to the exposed group after diagnosis. ^e^For the CDM, the exposed cohort included hospitalized patients from four hospitals without *M. pneumonia* infection and without autoimmune disease at the date of diagnosis of the index patient.

A total of 1,914,461 subjects were included and followed up from birth until 31/12/2017, using all included participants who were less than 18 years old on that date (**Figure 1**). The index date was defined as the date of first hospitalization due to *M. pneumoniae* infection. Individuals with underlying history of lower respiratory tract infection due to other pathogens (ICD-10 code J12X-J18X), respiratory and cardiovascular disorders specific to the perinatal period (ICD-10 code P20X-P29X), congenital malformations of the respiratory system (ICD-10 code Q30-Q34), chromosomal anomaly (ICD-10 code Q90-Q99), and lower respiratory tract infection due to solids and liquids (ICD-10 code J69X) were excluded. Individuals diagnosed as autoimmune diseases prior to the index date were also excluded. Lower respiratory tract infections due to other viral pathogens and infections due to ingestion of solids or liquids were also excluded, considering the potential inclusion of *M. pneumoniae* diagnoses or insufficient differentiation within these categories. Individuals in the exposed cohort were matched with unexposed controls at a 1:10 ratio based on birth year and sex using incidence density sampling. The unexposed cohort participants were randomly matched based on birth year and sex and had no *M. pneumoniae* infection or autoimmune diseases at the index patient’s diagnosis date.

### Exposure

The exposure in this study was defined as hospitalization due to *M. pneumoniae* infection, with relevant ICD-10 codes including J200 (acute bronchitis due to *M. pneumoniae*); J157 (pneumonia due to *M. pneumoniae*); B960 (*M. pneumoniae* as the cause of diseases classified elsewhere), or A493 (*Mycoplasma* infection, unspecified site) (14–16) (**Supplementary Table 2**). We referred to existing studies to define the exposure to enhance validity and minimize the possibility of misclassification (**Supplementary Tables 3**-**5**; literature search in **Supplementary Table 6**). Moreover, two pediatricians (Ha and Han) performed separate analyses to validate the predictive value of ICD-10 codes for the diagnosis of *M. pneumonia* infections (J200, J157, B960, or A493) by reviewing electronic medical records (EMRs) at Bundang CHA Medical Center and Kangnam Sacred Heart Hospital (17). The positive predictive values (PPVs) were 97.8% (95 CI, 95.8%–99.9%) and 97.5% (95% CI, 95.3%–99.7%), indicating that the diagnostic assessment used herein had high accuracy and was suitable for use in this analysis.

In addition, a Common Database Model (CDM) comparison, consisting of hospital-based data from four different hospitals that had data of patients with *M. pneumonia* infections and a matched unexposed cohort, was conducted to determine the robustness of the main results. These data were from different healthcare institutions and allowed large-scale distributed comparative effectiveness analysis (18). Evidence was generated using standard analytic tools by converting data to a CDM. For this analysis, patient-based retrospective cohort data were from four hospitals (Myongji Hospital [MJ], Kyung Hee University Hospital at Gangdong [KH], Bundang CHA Medical Center [CHA], Pusan National University Hospital [PS]). Heterogeneous data sources were standardized and analyzed using the analysis program that was distributed to these four institutions.

### Follow-up

All participants were followed from 01/01/2002 until the first diagnosis of an autoimmune disease, death, or the end of the study (31/12/2017), whatever happened first. The follow-up of unexposed individuals included the determination of a *M. pneumonia* infection later; an individual who was censored due to a *M. pneumonia* infection was transferred to the exposed group. The first year of follow-up was excluded from the analyses to reduce the probabilities of reverse causality and surveillance bias. The exposed individuals were followed for an average of 9.9 years (SD 3.5) and the unexposed cohort for 10.1 years (SD 3.4).

### Autoimmune diseases

Information on autoimmune diseases was from the NHIS database. The 41 autoimmune diseases (19, 20) were identified by *ICD-10* codes (**Supplementary Table 2**).

### Covariates

Data were examined for age, sex, follow-up time, birth residence, household income, calendar year/season at birth, any medical condition during the perinatal period, asthma comorbidity, and inpatient and outpatient hospital visits during the first year after study entry (21).. The most updated information before the index date was used for all analyses, and data on inpatient and outpatient visits were collected as a surrogate for use of medical resources during follow-up. Asthma was defined as one or more physician diagnoses 6 months prior to the index date, with one or more events of asthma exacerbation requiring hospitalization and/or systemic corticosteroids (22, 23). Use of systemic macrolide antibiotics infections during any month after the index date, duration of hospitalization, and use of oxygen therapy for treating *M. pneumoniae* during the first year after study entry were also assessed. We removed the first month after study entry from this calculation considering that exposed patients are likely to receive intensive medical care during the first month after *M. pneumoniae* infection.

### Statistical analysis

Conditional Cox models were used to estimate hazard ratios (HRs) with 95% CIs for developing an autoimmune disease due to previous *M. pneumonia* infection based on time after the index date. For this analysis, specific autoimmune diseases with fewer than 100 observed cases were not analyzed separately, but it was used in calculations of hazard ratios (HRs) for the corresponding main category. We performed stratified analyses by matching identifiers (birth year and sex) and adjusted for age group at index date (≤60 months or >60 months), time since index date (1 to <5 years or ≥5 years), calendar year of birth (2002–2003 or 2004–2005), birth residence (Seoul and metropolitan, city, or rural area), household income (by tertiles, low, middle, or high), calendar season at birth date (spring, summer, fall, or winter), any medical condition during the perinatal period (yes or no), asthma comorbidity (yes or no), inpatient hospital visits during the first year after study entry (yes or no), and outpatient hospital visits during the first year after study entry (≤13 or >13). All analyses were stratified by matching identifiers (birth year and sex) and adjusted for birth residence (Seoul/metropolitan, city, or rural area), household income (low, middle, or high), and perinatal history (disorder related to the length of gestation and fetal growth, birth trauma, infections specific to the perinatal period, congenital malformation/deformation, and chromosomal abnormalities). HRs were separately calculated for sex, age at the index date (≤60 months or >60 months), time since the index date (1–5 years or ≥5 years), calendar year of birth (2002–2003 or 2004–2005), birth residence (Seoul/metropolitan, city, or rural area), household income (low, middle, or high), the season of birth (spring, summer, fall, or winter), disorders related to any perinatal status (yes or no), history of asthma (yes or no), history of hospital admission (yes or no), and the number of outpatient visits (≤13 or >14, based on the median) during the first year after study entry. In the stratified analysis, information from the first month after study entry was not considered, as medical service utilization within that month was likely attributed to the exposure itself. Differences in HRs by variables were assessed by introducing an interaction term into the Cox models. The difference in log (HR) between strata was used to calculate the *z*-score and *P* value for this comparison. The absolute rate differences with 95% CIs were also determined for all associations.

Sensitivity analyses were performed to examine the effect of using a more stringent definition of *M. pneumonia* infection (ICD-10 code J157 alone), two or more and three or more autoimmune syndromes, individual autoimmune diseases, any of the major groups of autoimmune diseases (**Supplementary Table 2**) (20), or a more stringent definition of the outcome (use of medication such as non-steroidal anti-inflammatory drugs [NSAIDs], systemic steroid, intravenous immunoglobulin (24, 25), thyroid medications).

To determine the possible effects of reverse causality and surveillance bias, the analyses were repeated by excluding participants who were enrolled during the first 2 years (2002–2003) or during the first 5 years (2002–2007). In this analysis, the cumulative incidence curves of autoimmune diseases among participants with more than 1 year of follow-up were analyzed.

**Figure 2:**
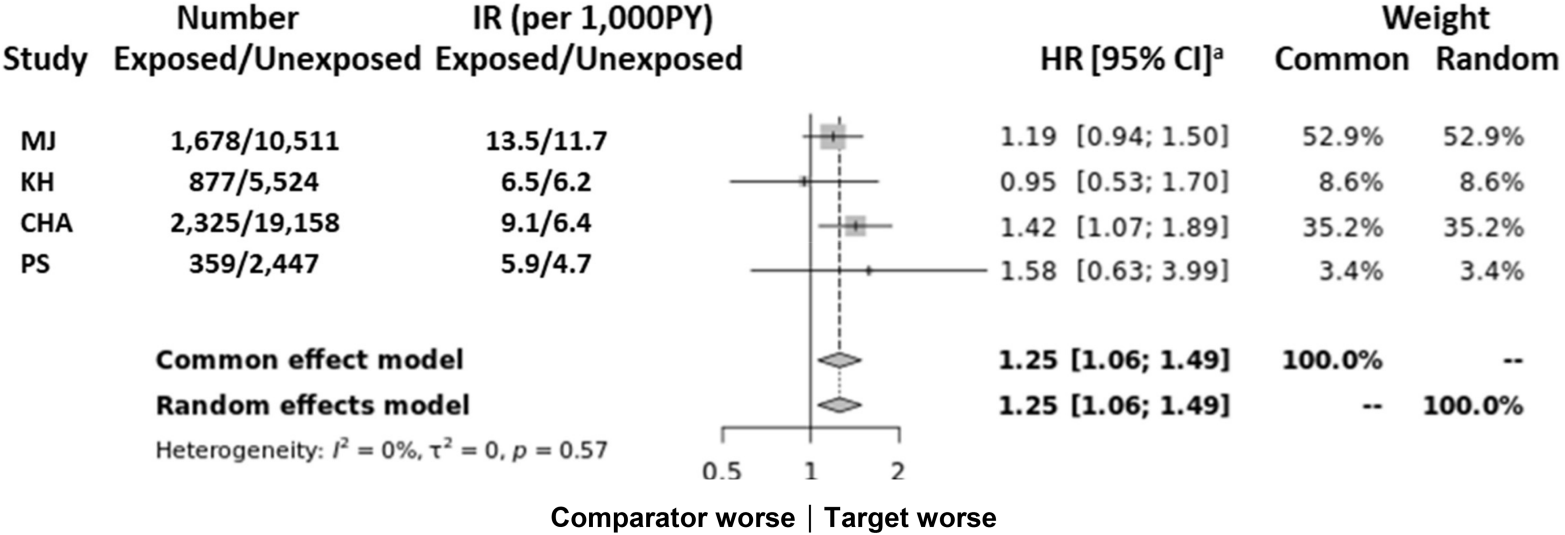
Multicenter analysis of results from the Common Data Model (**Supplementary Table 7**) of the association between *M. pneumonia*-related hospitalization and autoimmune disease. CHA, Bundang CHA Medical Center; CI, confidence interval; KH, Kyung Hee University Hospital at Gangdong; MJ, Myongji Hospital; N, number; PS, Pusan National University Hospital; PY, per year. ^a^The first year of follow-up was excluded from all analyses.

Furthermore, we performed an analysis using the CDM to assess the reliability of our results. We utilized four hospital-based cohorts that had been transformed into the OMOP-CDM (Observational Medical Outcomes Partnership Common Data Model) format. This format enabled the utilization of deidentified patient data extracted from EMRs. The hospitals included Myongji Hospital (MJ), Kyung Hee University Hospital at Gangdong (KH), CHA University Bundang CHA Medical Center (CHA), and Pusan National University Hospital (PS). This allowed for the multicenter analysis of disparate databases. This consisted of the following information: person, drug exposure, drug era, condition occurrence, condition error, observation period, observation, procedure occurrence, visit occurrence, death, drug cost, procedure cost, location, provider, organization, care site, payment plan period, and cohort (26). The analysis period and total number of patients were 2003 to 2020 and 880,392 from MJ, 2006 to 2017 and 822,183 from KH, 2006 to 2019 and 2,363,386 from CHA, and 2011 to 2018 and 1,753,001 from PS. In total, 5,818,962 patients were enrolled across all hospitals, consisting of 5,239 individuals in the exposed cohort (*M. pneumonia*-related hospitalization) and 37,640 in the unexposed cohort (**Figure 2**). All analyses were conducted in SAS statistical software version 9.4 (SAS Institute), and a two-sided *P* value below 0.05 was considered statistically significant.

### Ethics statement

The study was approved by the Institutional Review Board and Ethics Committees of Hallym University Kangnam Sacred Heart Hospital (IRB No. [2022-05-001]), Hallym University Kangdong Sacred Heart Hospital (IRB No. [2019-09-005]), and CHA University Bundang CHA Medical Center (IRB No. [2022-06-009]). The requirement for informed consent was waived because the study utilized a deidentified database open to the public in Korea.

## Results

### Baseline characteristics of the participants

All individuals who required hospitalization due to an initial *M. pneumonia* infection between 2002 and 2005 were identified (**Figure 1**). We excluded 348,741 individuals who met our predefined criteria, including those with a history of respiratory or cardiovascular complications during the intrauterine and perinatal period (n = 19,826), congenital malformations (n = 9,745), and chromosomal anomalies (n = 4,061). This left us with 52,805 children with *M. pneumonia* infections who met our criteria for inclusion in the study. We further excluded 2,868 children who had been diagnosed with autoimmune diseases, leaving us with 49,937 individuals in the exposed cohort. For the unexposed cohort, we identified individuals who had not been hospitalized for *M. pneumonia* infections and who met our inclusion criteria for population matching. Overall, there were 49,937 participants in the exposed cohort and 499,370 in the matched unexposed cohort.

The median age at diagnosis of *M. pneumoniae* infection was 3.67 years, and 48.54% of all patients were men (**Table 1**). Compared with the unexposed cohort, the exposed cohort had more inpatient and outpatient hospital visits and a higher rate of asthma comorbidity. The incidence of *M. pneumoniae* infection was greatest (0.8%) in children who were 2 and 3 years old and decreased steadily with age (**Supplementary Figure 1**). Trends in *M. pneumoniae* infection-related hospitalization from 2002 to 2016, analyzed using our database, are presented in **Supplementary Figure 2**. The variations in prevalence might be explained by the *M. pneumonia* epidemics in Korea during specific periods (27).

**Table 1:** Characteristics of the study cohorts.

Characteristic	Population-matched cohort, n (%)	
	Exposed cohort	Matched unexposed cohort
Participants, N	49,937	499,370
Follow-up time, mean (SD), y	9.9 (3.5)	10.1 (3.4)
Outcome date, mean (SD), y^a^	5.6 (3.2)	5.7 (3.2)
Male, N (%)	24,237 (48.5)	242,370 (48.5)
Age at index date, median years (IQR)	3.7 (2.1–6.4)	3.7 (2.1–6.4)
Age group, N (%)		
≤60 months old	32,477 (65.0)	324,770 (65.0)
>60 months old	17,460 (35.0)	174,600 (35.0)
Birth residence, N (%)		
Seoul/metropolitan	26,031(52.1)	264,616 (53.0)
City	18,357 (36.8)	184,950 (37.0)
Rural	5,549 (11.1)	49,678 (10.0)
Missing	0 (0.0)	126 (0.0)
Income tertile^b^, N (%)		
1 (lowest)	6,262 (12.5)	56,086 (11.2)
2 (middle)	32,650 (65.4)	320,158 (64.1)
3 (high)	9,304 (18.6)	103,912 (20.8)
Missing	1,721 (3.5)	19,214 (3.9)
Calendar year at birth, N (%)		
2002–2003	26,543 (53.2)	265,430 (53.2)
2004–2005	23,394 (46.9)	233,940 (46.9)
Calendar season of birth, N (%)		
Spring	13,062 (26.2)	133,398 (26.7)
Summer	11,560 (23.2)	116,931 (23.4)
Fall	12,482 (25.0)	120,825 (24.2)
Winter	12,833 (25.7)	128,216 (25.7)
Inpatient hospital visits during the first year after study entry^c^, N (%)		
None	41,117 (82.3)	472,765 (94.7)
≥1	8,820 (17.7)	26,605 (5.3)
Outpatient hospital visits during the first year after study entry, mean (SD)	19.1 (12.7)	15.0 (11.6)
Perinatal status		
Fetal growth and development disorder^d^, N (%)		
Yes	302 (0.6)	2,274 (0.5)
No	49,635 (99.4)	497,096 (99.5)
History of birth injury, N (%)		
Yes	97 (0.2)	709 (0.1)
No	49,840 (99.8)	498,661(99.9)
Infections specific to the perinatal period, N (%)		
Yes	2,930 (5.9)	24,627 (4.9)
No	47,007 (94.1)	474,743 (95.1)
Congenital malformation, N (%)		
Yes	6,860 (13.7)	55,391 (11.1)
No	43,077 (86.3)	443,979 (88.9)
Asthma comorbidity^e^, N (%)		
Yes	10,917 (21.9)	90,189 (18.1)
No	39,020 (78.1)	409,181 (81.9)
Specific diagnosis of *M pneumoniae* infection, N (%)		
Acute bronchitis due to *M pneumoniae* (J200)	5,743 (11.5)	
Pneumonia due to *M pneumoniae* (J157)	47,133 (94.4)	
*M pneumoniae* as the cause of diseases classified elsewhere (B960)	252 (0.5)	
*Mycoplasma* infection, unspecified site	853 (1.7)	
Use of antibiotics^f^, N (%)		
Yes	49,547 (99.2)	
No	390 (0.8)	
Use of systemic corticosteroid^g^, N (%)		
Yes	10,357 (20.7)	
No	39,580 (79.3)	
Oxygen therapy^g^, N (%)		
Yes	1,484 (3.0)	
No	48,453 (97.0)	
Length of hospitalization^h^, N (%)		
1–6 days	24,197 (48.5)	
≥7 days	25,740 (51.5)	

IQR.

a The period between the index date and the occurrence of the outcome among children who experienced the outcome.

b Income was categorized into tertiles based on the amount of insurance co-payment.

c The first month after study entry was removed from this calculation.

d Complications during the intrauterine and perinatal period that were diagnosed 3 months prior to the index date (i.e., diagnosis date of the exposed participant or the diagnosis date of the index participant for the matched unexposed participant).

e Defined as one or more physician diagnosis during 6 months prior to the index date, with 1 or more event of asthma exacerbation requiring hospitalization or systemic corticosteroids.

f Prescriptions of systemic macrolide antibiotics for treating *M. pneumoniae* infections during any month after the index date.

g Events during 1 month after the index date.

h The median length of hospitalization was 6 days.

### Relationship between hospitalization due to *M. pneumoniae* infection and increased risks to autoimmune diseases

Elevated HRs of autoimmune diseases in the exposed group with history of hospitalization due to *M. pneumoniae* infection was shown by the Cox model with adjustment of multiple confounders, compared with the unexposed group (**Table 2**). During a mean follow-up time of 10 years, we identified newly diagnosed autoimmune diseases in 3295 individuals in the exposed cohort (incidence rate: 66.5 per 10,000 person-years; 95% CI: 64.3–68.8) and in 26,145 individuals in the unexposed cohort (incidence rate: 52.3 per 10,000 person-years; 95% CI: 51.7–52.9). This corresponded to an absolute difference of 14.3 per 10,000 person-years (95% CI: 11.9–16.6), with an HR of 1.26 (95% CI: 1.215–1.308).

**Table 2:** Risk of autoimmune disease in the exposed cohort (patients hospitalized with *M. pneumoniae* infection) relative to the matched unexposed cohort.

	Cases/accumulated person-years × 10,000 (incidence rate/10,000 person-years)		Absolute rate difference/10,000 person-Years (95% CI)	Hazard ratio (95% CI)^b^	*P* value^c^
	Exposed cohort	Matched unexposed cohort			
All	3,295/49.5 (66.5)	26,145/499.7 (52.3)	14.27 (11.91–16.64)	1.26 (1.215-1.308)	
Sex					
Female	1,731/25.2 (68.7)	13,711/255 (53.9)	14.92 (11.56–18.28)	1.257 (1.195-1.323)	0.9036
Male	1,564/24.3 (64.4)	12,434/245 (50.7)	13.61 (10.3–16.92)	1.264 (1.198-1.333)	
Age group at index date					
≤60 months	2,578/38.0 (67.8)	20,945/380 (54.6)	13.3 (10.58–16.02)	1.228 (1.178-1.280)	**0.0085**
>60 months	717/11.5 (62.4)	5,200/119 (44.9)	17.52 (12.8–22.24)	1.391 (1.285-1.507)	
Time since index date					
1 to <5 years	2,562/37.7 (68.0)	20,760/403 (54.6)	13.33 (10.59 –16.06)	1.232 (1.181-1.284)	**0.0211**
≥5 years	733/11.8 (62.0)	5,385/97 (45.2)	16.87 (12.21–21.52)	1.373 (1.269-1.485)	
Calendar year of birth					
2002–2003	1,940/26.1 (74.3)	14,970/264 (56.7)	17.63 (14.2–21.06)	1.298 (1.237-1.362)	0.0726
2004–2005	1,355/23.4 (57.9)	11,175/236 (47.4)	10.55 (7.35–13.76)	1.212 (1.144-1.283)	
Birth residence					
Seoul and metropolitan	1,828/25.7 (71.1)	14,385/264 (54.4)	16.64 (13.26–20.02)	1.293 (1.231-1.358)	0.1547
City	1,145/18.3 (62.6)	9,362/185 (50.5)	11.96 (8.2–15.73)	1.224 (1.150-1.303)	
Rural	322/5.5 (58.3)	2,398/50 (48.2)	10.59 (3.91–17.26)	1.213 (1.075-1.369)	
Income tertile					
Low	387/6.2 (62.2)	2,844/56 (50.6)	11.63 (5.14–18.13)	1.213 (1.091-1.349)	0.7149
Middle	2,179/32.4 (67.2)	16,808/320 (52.5)	14.73 (11.8–17.66)	1.272 (1.217-1.331)	
High	627/9.2 (68.1)	5,604/104 (54.0)	14.27 (8.75–19.79)	1.252 (1.152-1.359)	
Calendar season at birth date					
Spring	891/13 (69.0)	7,234/133 (54.3)	15.74 (11.11–20.37)	1.253 (1.167-1.344)	0.75
Summer	747/11 (65.3)	6,073/117 (51.9)	13.22 (8.38–18.06)	1.258 (1.164-1.359)	
Fall	796/12 (63.8)	6,066/121 (50.0)	14.89 (10.09–19.7)	1.254 (1.163-1.352)	
Winter	861/13(67.9)	6,772/128 (52.8)	12.38 (7.82–16.95)	1.275 (1.186-1.370)	
Any medical condition during the perinatal period ^d^					
Yes	731/9.4 (77.8)	4,952/77.6 (63.8)	13.92 (8.02–19.84)	1.213 (1.121-1.312)	0.2539
No	2564/40.1 (63.9)	21,193/422.1 (50.2)	11.15 (11.15–16.28)	1.274 (1.222-1.328)	
Asthma comorbidity^e^					
Yes	619/9.4 (65.7)	3,967/77.3 (51.3)	14.38 (8.96–19.80)	1.272 (1.166-1.386)	0.9007
No	2,676/40.1 (66.7)	22,178/422.3 (52.5)	14.23 (11.61–16.85)	1.260 (1.209-1.312)	
Inpatient hospital visits during the first year after study entry^f^					
Yes	783/9.2 (85.1)	2,181/28.5 (76.6)	8.58(1.81–15.35)	1.110 (1.022-1.206)	**0.0488**
No	2,512/40.3 (62.3)	23,964/471.2 (50.9)	11.92 (9.38–14.46)	1.217 (1.167-1.269)	
Outpatient hospital visits during the first year after study entry n^f^					
≤13	923/17.0 (54.3)	11,596/251.1 (46.2)	8.11(4.51–11.71)	1.191 (1.113-1.274)	0.9088
>13	2,365/32.4 (73.1)	14,099/228.4 (61.7)	11.33(8.21–14.44)	1.172 (1.121-1.226)	

a Results not shown if the number of subjects was less than 10.

b Cox models were stratified by matching identifiers (birth year and sex) and adjusted for birth residence (Seoul/metropolitan, city, or rural area), household income (low, middle, or high), perinatal history (disorders related to fetal growth and development, birth injury, congenital malformations), and prescription duration of systemic macrolide antibiotics. The first year of follow-up was excluded from all analyses.

c P value was from an interaction test by incorporating an interaction term into the Cox model.

d Complications during the intrauterine and perinatal period that was diagnosed 3 months prior to the index date (i.e., the date of diagnosis of an exposed participant or the date of diagnosis of the index participant with the matched unexposed participant); history of fetal growth and development disorders, birth injury, infections specific to the perinatal period, and congenital malformations.

e Defined as one or more physician diagnoses during 6 months prior to the index date, with one or more events of asthma exacerbation requiring hospitalization or systemic corticosteroids.

f Removed the first month after study entry from this calculation.

Bold values indicate statistical significance at the p < 0.05 level.

Sex, calendar year of birth, asthma comorbidity, perinatal status, and the number of outpatient visits had no significant effects on the HR for autoimmune disease. However, the HR was less in patients infected at an age of 60 months or less (HR: 1.228; 95% CI: 1.178–1.280 *vs.* HR: 1.391; 95% CI: 1.285–1.507; *P* for interaction = 0.0085) and in patients with an index date of 1 to 5 years (HR: 1.232; 95% CI: 1.181–1.284 *vs.* HR: 1.373; 95% CI: 1.269–1.485; *P* for interaction = 0.021). The HR was marginally smaller for those with a history of hospital admission during the first year after study entry (HR: 1.10; 95% CI: 1.022–1.206 *vs.* HR: 1.217; 95% CI: 1.167–1.269; *P* for interaction = 0.049).

**Figure 3:**
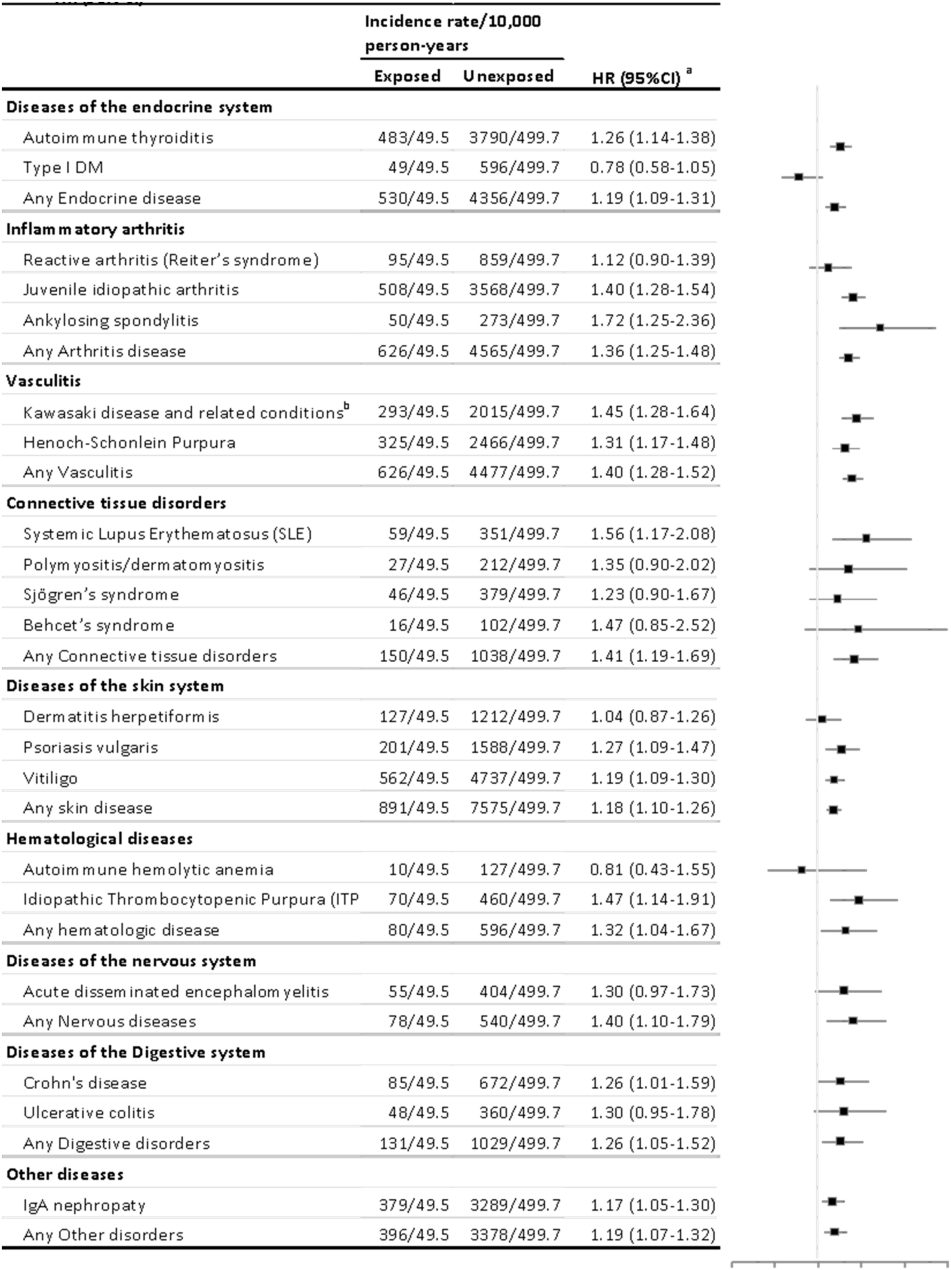
Risk estimates for the association of *M. pneumonia*-related hospitalization with different types of autoimmune diseases. HR, hazard ratio. Autoimmune diseases with fewer than 100 cases were not analyzed separately but were used for calculations of hazard ratios (HRs) for the main categories. Cox models were stratified by matching identifiers (birth year and sex) and adjusted for birth residence (Seoul/metropolitan, city, or rural area), household income (low, middle, or high), and perinatal history (disorder related to length of gestation and fetal growth, birth trauma, infections specific to the perinatal period, congenital malformation/deformation, and chromosomal abnormalities). The first year of follow-up was excluded from all analyses. ^a^Autoimmune diseases with fewer than 100 cases were not analyzed separately but were included in calculations of hazard ratios (HRs) for the main categories. ^b^Includes autoimmune vascular disorders, such as polyarteritis nodosa and Churg–Strauss syndrome.

*M. pneumoniae* infection was also associated with elevated risks in each of the nine major groups of autoimmune diseases (**Figure 3**). The population-matched analysis indicated that these associations were especially notable for four groups of autoimmune diseases. Several associations between *M. pneumoniae* infection and several autoimmune diseases were notable, including autoimmune thyroiditis (HR 1.258, 95% CI 1.142–1.386), juvenile idiopathic arthritis (HR 1.403, 95% CI 1.276–1.543), ankylosing spondylitis (HR 1.72, 95% CI 1.253–2.361), Kawasaki disease (HR 1.449, 95% CI 1.280–1.640), Henoch-Schonlein purpura (HR 1.314, 95% CI 1.168–1.478), systemic lupus erythematosus (SLE) (HR 1.559, 95% CI 1.169–2.077), psoriasis vulgaris (HR 1.268, 95% CI 1.092–1.472), vitiligo (HR 1.185, 95% CI 1.085–1.295), idiopathic thrombocytopenic purpura (ITP) (HR 1.474, 95% CI 1.14–1.907), Crohn’s disease (HR 1.26, 95% CI 1.006–1.586), and IgA nephropathy (HR 1.167, 95% CI 1.048–1.299). Among these, the risk for SLE was the highest in the exposed cohort, and no significantly lower risks were observed in any of the other autoimmune diseases.

### Effects of healthcare history, age at infection, and antibiotic use on autoimmune diseases hospitalization due to *M. pneumoniae* infection

We examined the effects of use of antibiotics, oxygen therapy, and hospital visits on the association between hospitalization due to *M. pneumoniae* infection and autoimmune disease. The association was influenced by the prescription of antibiotics from 1 month before to 1 month after the index date, whereas the significance interaction was marginal. Oxygen therapy and frequency of outpatient visits within a month after the index date did not show an effect (**Supplementary Table 7**).

### Multiple sensitivity analyses

The CDM comparison corroborated the observed associations (**Figure 2**) with higher estimates noted for autoimmune diseases (HR: 1.25; 95% CI: 1.06–1.49) although there were differences in statistical values among the hospitals.

The results of further sensitivity analysis yielded consisted results (**Table 3**). The association of hospitalization for *M. pneumoniae* infection with autoimmune disease was stronger for those with two or more autoimmune syndromes (HR: 1.506; 95% CI: 1.314–1.727; P for difference = 0.0011) and with three or more autoimmune syndromes (HR: 1.789; 95% CI: 1.178–2.716; *P* for difference <0.001) (**Table 3**). The sensitivity analysis also indicated greater risk when excluding cases diagnosed 2 years after study entry or 5 years after study entry; when exposure was defined more stringently (only ICD-10 code J157); and when the outcome was defined more stringently (diagnosis and treatment for autoimmune disease). In addition, when participants were restricted as individuals followed up for more than 5 years after entry, the exposed group (history of hospitalization for *M. pneumoniae* infection) had a higher cumulative incidence of autoimmune diseases compared with their matched unexposed group across the follow-up period (**Supplementary Figure 3**).

**Table 3:** Sensitivity analysis of the association between *M. pneumonia*-related hospitalization and autoimmune disease.

	Cases/accumulated person-years × 10,000 (incidence rate/10,000 person-years)		Absolute rate difference/10,000 person-years (95% CI) ^a^	Hazard ratio (95% CI)
Sensitivity analysis (reference group)	Exposed individuals	Matched unexposed individuals		
Multiple autoimmune syndromes (any syndrome)				
≥2	245/6.9 (35.5)	1,619/70 (23.1)	11.96 (7.42–16.50)	1.506 (1.314–1.727)
≥3	26/7.0 (3.7)	150/70 (2.1)	1.54 (0.99–2.09)	1.789 (1.178–2.716)
Lag-time				
2 years after study entry was excluded	2,918/44.9 (64.9)	23,362/453 (51.6)	13.41 (10.96–15.86)	1.251 (1.203–1.301)
5 years after study entry was excluded	1,924/31.2 (61.6)	15,721/314 (50.1)	11.58(8.72–14.45)	1.223 (1.166–1.284)
Restricted exposure^b^ (any of 4 ICD codes)				
ICD-10 code J157	3124/46.9 (66.7)	26145/473 (52.3)	14.27 (11.85–16.7)	1.27 (1.22–1.31)
More stringent definition of the outcome (diagnosis alone)				
Diagnosis and medication	1,541/50.7 (30.4)	11,683/509 (22.9)	7.47 (5.90–9.05)	1.32 (1.25–1.39)

a Cox models were stratified by matching identifiers (birth year and sex) and adjusted for birth residence (Seoul/metropolitan, city, or rural area), household income (low, middle, or high), perinatal history (disorders related to fetal growth and development, birth injury, congenital malformations), and prescription duration of systemic macrolide antibiotics. The first year of follow-up was excluded from all analyses.

b P value was from an interaction test by incorporating an interaction term into the Cox model.

## Discussion

In this large-scaled population-based cohort study, we observed a higher risk of autoimmune disease following hospitalization for *M. pneumoniae* infection. This association was particularly pronounced among patients who were older at the time of admission (as determined by diagnosis data) and whose index date was beyond 60 months. We found that the association persisted regardless of hospitalization duration, oxygen therapy, and antibiotic use. To the best of our knowledge, this is the first study to examine the relationship of hospitalization for *M. pneumoniae* infection with 19 distinct autoimmune in a large pediatric population, using a population-based comparison. By shedding light on this possible link, our study underscores the need for continued research regarding the potential link between severe *M. pneumoniae* infection and autoimmune and immune-mediated diseases (28, 29).

Our findings also suggest that degree of association differed for the various autoimmune diseases examined in our study. For instance, the hazard ratio was 1.40 for juvenile idiopathic arthritis and 1.26 for autoimmune thyroid disease. These differences may reflect variations in the prevalence and pathogenicity of different autoimmune diseases in children Moreover, our results are consistent with previous studies that have reported associations between *M. pneumoniae* infection and autoimmune diseases such as Guillain–Barre’s syndrome (GBS) (30, 31) and polyarthritis (32). However, these previous studies were limited by a cross-sectional design, which precluded causal inferences. While our study provides compelling evidence for a potential link between severe infection and autoimmune and immune-mediated diseases, it is important to acknowledge that further research is needed to fully understand the mechanisms underlying this association (28).

Our findings are consistent with previous evidence that reported links of severe cases of *M. pneumoniae* infection and related respiratory diseases with immune system dysfunction and suggest that autoimmune reactions may contribute to some of the extrapulmonary complications in patients who have these infections (33). Previous evidence suggests that this may be attributed to immune responses related to cytokine activation and immune cell reaction due to severe infection. For example, inflammatory reflexes precipitated by *M. pneumoniae* infection might activate the sympathetic nervous system, possibly leading to impaired immune function or a widespread inflammatory response. Moreover, the adhesin proteins of *M. pneumoniae* (P1, P30, and P116) enable the adhesion of this pathogen to host epithelial cells, and this leads to the production of antibodies that target cells in the brain, liver, kidney, smooth muscle, and lungs that could induce autoimmune disorders in the affected organs (34, 35). The release of cytokines and inflammatory mediators during *M. pneumoniae* infection can lead to worse disease outcomes and overactivation of the immune system (1, 36). This supports our finding that patients hospitalized for *M. pneumoniae* infection were more likely to develop autoimmune and immune-mediated diseases involving different organs and multiple autoimmune syndromes. This might also explain the stronger association of infection with an autoimmune disease in children exposed after the age of 60 months, whose clinical course is more severe. Immune reactions induced by *M. pneumoniae* infections might occur because of the amino-acid sequence homology of mycoplasmal adhesins with antigens (P1, P30, and perhaps P65 and P116) in diverse human tissues and the formation of immune complexes (37). *M. pneumoniae* infections can activate T cells and increase B-cell proliferation, and these play important roles in the pathogenesis of extrapulmonary manifestations during pneumonia and autoimmune diseases (3). However, another study found no significant difference in the age of occurrence of extrapulmonary symptoms possibly related with immune-mediated reactions between *M. pneumoniae* infection and respiratory infections caused by *M. pneumoniae* in children. Caution is warranted in interpreting this finding due to the inherent limitations of epidemiological studies (38).

The strengths of our study are that we used a population-based cohort design and performed a complete follow-up of nearly 50,000 patients who were diagnosed with *M. pneumoniae* infections during a 10-year period. Furthermore, the large sample size allowed us to perform several sensitivity analyses, so we analyzed the effect of age at exposure and the use of multiple outcome measures. The availability of detailed sociodemographic and medical information also allowed us to control for a large number of potential confounders.

This study has several limitations. First, there may have been some surveillance bias. To address this issue, we performed sensitivity analyses using extended lag times, measured different outcomes, and adjusted for estimated levels of medical surveillance. Second, we relied on diagnoses of infection registered in the NHIS. This could have been an underestimation if infection occurred as a comorbidity of a different severe condition, if there was delayed identification of the pathogen, or if there was a missed diagnosis. Furthermore, since exposures included inpatient cases, the associations between Mycoplasma pneumoniae infection and autoimmune diseases may be limited to severe cases and may not be applicable to non-hospitalized infections. Third, since this is an observational retrospective cohort study, we could not exclude potential confounders that may affect the causal relationship because of the nature of this data. As an example, changes in epidemics over time such as age, sex, and peak season that might influence the pathogenesis of *M. pneumoniae* infection were not assessable. Furthermore, unmeasured factors such as unreported health conditions preceding an autoimmune disease (39), alterations in health-related behaviors, or the use of certain medications should be addressed in future studies. Fourth, although we identified a stronger association for older children, we did not assess the association between *M. pneumoniae* infections with autoimmune diseases that occurred later in life.

## Conclusions

This nationwide population-based cohort study may indicate that exposure to *M. pneumoniae* infection requiring hospitalization may be associated with an increase in subsequent diagnoses of autoimmune diseases. Although our findings provide insight into the relationship between hospitalizations due to *M. pneumonia* infection and the increased occurrence of autoimmune disease, further studies are needed to better understand the underlying mechanisms.

## Data availability statement

The original contributions presented in the study are included in the article/**Supplementary Material**. Further inquiries can be directed to the corresponding author.

## Ethics statement

The studies involving humans were approved by Institutional Review Board and Ethics Committees of Hallym University Kangnam Sacred Heart Hospital. The studies were conducted in accordance with the local legislation and institutional requirements. Written informed consent for participation was not required from the participants or the participants’ legal guardians/next of kin in accordance with the national legislation and institutional requirements.

## Author contributions

MH and SC contributed to the conception of the work, design, analysis, interpretation of the data, and final approval of the version to be published; EH mainly drafted the manuscript and final approval of the version to be published; H-SB and YS contributed to the design of the work, interpretation of the data, drafting of the work, and final approval of the version to be published; HS-B, and YS contributed to the interpretation and analysis of the data, revising it critically for important intellectual content and final approval of the version to be published; HC and MH contributed to data collection, and analysis for the work, revising it critically for important intellectual content and final approval of the version to be published.
