# Supplementary material for: Investigating the occurrence of autoimmune diseases among children and adolescents hospitalized for Mycoplasma pneumoniae infections
Source: Front Immunol. 2023 Dec 5;14:1165586. doi: 10.3389/fimmu.2023.1165586 (PMC10732509; doi:10.3389/fimmu.2023.1165586)
Supplement: Supplementary file 1 [file DataSheet_1.docx]

**Supplementary Table 1.** Checklist of recommendations for REporting of studies Conducted using Observational Routinely-collected health Data (RECORD)

**Supplementary Table 2.**  MeSH terms and ICD-10 codes used to define exposures and outcomes

**Supplementary Table 3.** Previous case reports of *M. pneumoniae* infections and autoimmune diseases in children.

**Supplementary Table 4.** Previous cross-sectional and non-controlled observational studies of *M. pneumoniae* infection and autoimmune disease in children

**Supplementary Table 5.** Previous Case-Control studies of *M. pneumoniae* infection and autoimmune diseases in children

**Supplementary Table 6.** Terms used for searching PubMed.

**Supplementary Table 7.**Risk of autoimmune disease in the exposed cohort (patients admitted with *M. pneumoniae* infection) based on different indicators of infection

**Supplementary Table 8.** Data used for the meta-analysis (Figure 3) of the association between *M. pneumoniae* infection and autoimmune diseases from the Common Data Model (CDM)

**Supplementary Table 9.** Characteristics of participants included in the Common Data Model (CDM) analysis at each hospital

**Supplementary Figure 1.** Incidence of *M. pneumoniae* infections in children of different ages during the study period

**Supplementary Figure 2.** Trends in *M. pneumoniae* infection-related hospitalization from 2002 to 2016

**Supplementary Figure 3.** Cumulative incidence rates of autoimmune diseases for participants in the exposed group (history of hospitalization due to M. pneumoniae infection) and the matched unexposed group

**Supplementary Table 1. Checklist of recommendations for REporting of studies Conducted using Observational Routinely-collected health Data (RECORD)**

|  | Item No | Recommendation | Reported |
| --- | --- | --- | --- |
| Title and abstract | 1 | (a) Indicate the study's design with a commonly used term in the title or the abstract | Abstract |
|  |  | (b) Provide in the abstract an informative and balanced summary of what was done and what was found | Abstract |
| **Introduction** |  |  |  |
| Background /rationale | 2 | Explain the scientific background and rationale for the investigation being reported | Introduction |
| Objectives | 3 | State specific objectives, including any prespecified hypotheses | Introduction |
| **Methods** |  |  |  |
| Study design | 4 | Present key elements of study design early in the paper | Methods: Study design and Figure 1 |
| Setting | 5 | Describe the setting, locations, and relevant dates, including periods of recruitment, exposure, follow-up, and data collection | Methods: Study design |
| Participants | 6 | (a) Give the eligibility criteria, and the sources and methods of selection of participants. Describe methods of follow-up | Methods: Study design and Figure 1 |
|  |  | (b) For matched studies, give matching criteria and number of exposed and unexposed | Methods: Study design and Figure 1 |
| Variables | 7 | Clearly define all outcomes, exposures, predictors, potential confounders, and effect modifiers. Give diagnostic criteria, if applicable | Methods: Exposures, Autoimmune Diseases, Covariates |
| Data sources /measurement | 8 | For each variable of interest, give sources of data and details of methods of assessment (measurement). Describe comparability of assessment methods if there is more than one group | Methods: Covariates |
| Bias | 9 | Describe any efforts to address potential sources of bias | Methods: Covariates; Statistical analysis; |
| Study size | 10 | Explain how the study size was arrived at | Methods: Study design and Figure 1 |
| Quantitative variables | 11 | Explain how quantitative variables were handled in the analyses. If applicable, describe which groupings were chosen and why | Methods: Statistical analysis |
| Statistical methods | 12 | (a) Describe all statistical methods, including those used to control for confounding | Methods: Covariates; Statistical analysis |
|  |  | (b) Describe any methods used to examine subgroups and interactions | Methods: Statistical analysis |
|  |  | (c) Explain how missing data were addressed | Statistical analysis and Table 1 |
|  |  | (d) If applicable, explain how loss to follow-up was addressed | Methods: Follow-up |
|  |  | (e) Describe any sensitivity analyses | Methods: Study design; Statistical analysis |
| **Results** |  |  |  |
| Participants | 13 | (a) Report numbers of individuals at each stage of the study, e.g., numbers potentially eligible, examined for eligibility, confirmed eligible, included in the study, completing follow-up, and analyzed | Results, Figure 1 |
|  |  | (b) Give reasons for non-participation at each stage | Figure 1 |
|  |  | (c) Consider use of a flow diagram | Figure 1 |
| Descriptive  data | 14 | (a) Give characteristics of study participants (e.g., demographic, clinical, social) and information on exposures and potential confounders | Results, Participants and covariates, Table 1 |
|  |  | (b) Indicate number of participants with missing data for each variable of interest | Results, Table 1 |
|  |  | (c) Summarize follow-up time (e.g., average and total amount) | Methods: Follow-up |
| Outcome data | 15 | Report numbers of outcome events or summary measures over time | Results, Table 2, and Figure 2 |
| Main results | 16 | (a) Give unadjusted estimates and, if applicable, confounder-adjusted estimates and their precision (e.g., 95% confidence interval). Make clear which confounders were adjusted for and why they were included | Results, Table 2, and supplement table 3 |
|  |  | (b) Report category boundaries when continuous variables were categorized | Not applicable |
|  |  | (c) If relevant, consider translating estimates of relative risk into absolute risk for a meaningful time period | Not applicable |
| Other analyses | 17 | Report other analyses done, e.g., analyses of subgroups and interactions, and sensitivity analyses | Figure 2, Table 3 |
| **Discussion** |  |  |  |
| Key results | 18 | Summarize key results with reference to study objectives | Discussion |
| Limitations | 19 | Discuss limitations of the study, taking into account sources of potential bias or imprecision. Discuss both direction and magnitude of any potential bias | Discussion |
| Interpretation | 20 | Give a cautious overall interpretation of results considering objectives, limitations, multiplicity of analyses, results from similar studies, and other relevant evidence | Discussion |
| Generalizability | 21 | Discuss the generalizability (external validity) of the study results | Discussion |
| Other information |  |  |  |
| Funding | 22 | Give the source of funding and the role of the funders for the present study and, if applicable, for the original study on which the present article is based | Article Information |

**Supplementary Table 2.  MeSH terms and ICD-10 codes used to define exposures and outcomes**

|  | **Disease** |  | **MeSH Term** | **ICD-10 code** |  |
| --- | --- | --- | --- | --- | --- |
|  | All mycoplasma pneumoniae infection related disease | Acute bronchitis due to *Mycoplasma pneumoniae* | Mycoplasma; "mycoplasma*"[All Fields] | J200 |  |
|  |  | Pneumonia due to *Mycoplasma pneumoniae* |  | J157 |  |
|  |  | *Mycoplasma pneumoniae* as the cause of diseases classified elsewhere |  | B960 |  |
|  |  | *Mycoplasma* infection |  | A493 |  |
|  | Disease of endocrine system | Diabetes mellitus, insulin dependent | Diabetes mellitus, type 1 | E10 |  |
|  |  | Autoimmune thyroid disease | Thyroiditis, autoimmune | E03.5, E03.9, E05.0, E05.5, E05.9, E06.3, E06.5 |  |
|  |  | Addison’s disease | Addison Disease | E27.1, E27.2 |  |
|  |  | Autoimmune polyglandular syndrome | Polyendocrinopathies, autoimmune; Autoimmune polyendocrinopathy syndrome type 1 | E31.0 |  |
|  | Inflammatory arthritis | Reactive arthritis (Reiter syndrome) | Arthritis, reactive | M02.3, M02.8, M02.9, M05, M06, M08.0 |  |
|  |  | Rheumatoid arthritis | Arthritis, juvenile | M08.1, M08.2, M08.3. M08.4 |  |
|  |  | Ankylosing spondylitis | Spondylitis, ankylosing | M45 |  |
|  | Vasculitis | Kawasaki disease and related condition | Mucocutaneous Lymph Node Syndrome; Polyarteritis Nodosa; Churg-Strauss Syndrome | M30 |  |
|  |  | Thrombotic microangiopathy | Thrombotic Microangiopathies | M31.1 |  |
|  |  | Wegener’s granulomatosis | Granulomatosis with Polyangiitis | M31.3 |  |
|  |  | Microscopic polyangiitis | Microscopic Polyangiitis | M31.7 |  |
|  |  | Henoch-Schonlein purpura | Vascular purpura | D69.0 |  |
|  |  | Giant cell arteritis/polymyalgia rheumatica | Giant Cell Arteritis; Polymyalgia Rheumatica | M35.3, M31.5, M31.6 |  |
|  | Connective tissue disorders | Systemic lupus erythematosus | Lupus erythematosus, systemic | M32 |  |
|  |  | Polymyositis/dermatomyositis | Polymyositis; Dermatomyositis | M33.0, M33.1, M33.2, M33.9 |  |
|  |  | Systematic sclerosis (scleroderma) | Scleroderma, systemic; Scleroderma, diffuse | M34 |  |
|  |  | Sjögren’s syndrome | Sjogren's Syndrome | M35.0 |  |
|  |  | Mixed connective tissue disease | Mixed Connective Tissue Disease | M35.1 |  |
|  |  | Behcet’s syndrome | Behcet Syndrome | M35.2 |  |
|  | Disease of skin system | Pemphigus vulgaris | Pemphigus | L10.0 |  |
|  |  | Bullous pemphigoid | Pemphigoid, bullous | L12 |  |
|  |  | Dermatitis herpetiformis | Dermatitis Herpetiformis | L13.0 |  |
|  |  | Psoriasis | Psoriasis | L40 |  |
|  |  | Alopecia areata | Alopecia Areata | L64 |  |
|  |  | Vitiligo | Vitiligo | L80 |  |
|  | Hematological diseases | Pernicious anemia | Anemia, pernicious | D51.0 |  |
|  |  | Autoimmune hemolytic anemia | Anemia, hemolytic, autoimmune | D59.0, D59.1 |  |
|  |  | Idiopathic thrombocytopenic purpura | Purpura, thrombocytopenic, idiopathic | D69.3 |  |
|  | Disease of nervous system | Acute disseminated encephalomyelitis | Encephalomyelitis, acute disseminated | G04 |  |
|  |  | Anti-NMDA receptor encephalitis | Anti-N-Methyl-D-Aspartate Receptor Encephalitis | G13.1 |  |
|  |  | Multiple sclerosis | Multiple Sclerosis; Multiple sclerosis, chronic progressive; Multiple sclerosis, relapsing remitting | G35 |  |
|  |  | Neuromyelitis optica and ADEM | Neuromyelitis Optica | G36 |  |
|  |  | Guillain-Barré syndrome | Guillain-Barre Syndrome | G61.0, G61.1, G61.8, G61.9 |  |
|  |  | Myasthenia gravis | Myasthenia Gravis | G70.0 |  |
|  | Disease of digestive system | Primary biliary cirrhosis | Liver cirrhosis, biliary | K74.3 |  |
|  |  | Crohn’s disease | Crohn Disease | K50 |  |
|  |  | Ulcerative colitis | Colitis, ulcerative | K51 |  |
|  |  | Coeliac disease | Celiac Disease | K90.0 |  |
|  | Others | Acute rheumatic fever and chorea | Rheumatic Fever; Chorea | I00, I01.0, I01.1, I01.2, I01.8, I01.9, I02.0, I02.9 |  |
|  |  | Sarcoidosis | Sarcoidosis; Sarcoidosis, pulmonary | D86 |  |
|  |  | IgA nephropathy | Glomerulonephritis, IgA | N00,N01,N03,N05 |  |

Abbreviations: ADEM, acute disseminated encephalomyelitis; ICD, International Classification of Diseases; NMDA, N-Methyl-D-aspartate.

**Supplementary Table 3. Previous case reports of *M. pneumoniae* infections and autoimmune diseases in children**

| **Author/year of publication** | **Type of study** | **Age/sex** | ***Mycoplasma pneumoniae* (MP) Serology** | **Clinical manifestations** | **Related autoimmune disease** |
| --- | --- | --- | --- | --- | --- |
| [Sakoulas et al. 2001](https://pubmed.ncbi.nlm.nih.gov/11368117/) ^1^ | Case report | 6 years/female | Positive serum MP IgM | Nausea, vomiting, cough and high fever | Brainstem and striatal encephalitis |
| [Chryssanthopoulos et al. 2001](https://pubmed.ncbi.nlm.nih.gov/11419512/) ^2^ | Case report | 7 years/male | Four-fold rise in titer of complement fixation for MP and open lung biopsy | Cough, fever (39.5°C) and malaise | Disseminated intravascular coagulation |
| [Wang et al. 2001](https://pubmed.ncbi.nlm.nih.gov/11430729/)^3^ | Case report | 11 years/male | Serological investigations suggestive of MP infection | Fever, cough, headache, dizziness skin rash | Kawasaki disease |
| [Ashtekar et al. 2003](https://pubmed.ncbi.nlm.nih.gov/12948332/)^4^ | Case report | 5 years/male | Pneumonia with pleural effusion, confirmed to be MP infection | Prolonged generalized tonic or tonic-clonic convulsions on 7th day of illness | Acute bilateral thalamic necrosis |
| [Hsueh et al. 2004](https://pubmed.ncbi.nlm.nih.gov/15493737/)^5^ | Case report | 6 years/female | Serologically proven MP infection | Ophthalmoplegia, ataxia, and areflexia | Miller Fisher syndrome |
| [Aydin et al. 2005](https://pubmed.ncbi.nlm.nih.gov/15607608/)^6^ | Case report | 4 years/male | Antibody titers against MP increased in serum and CSF | Seizures and transient amaurosis after febrile infection | Acute disseminated encephalomyelitis |
| [Arakawa et al. 2005](https://pubmed.ncbi.nlm.nih.gov/16122631/)^7^ | Case report | 11 years/male | Anti-MP IgM antibody significantly elevated | Severe paralysis and respiratory insufficiency | Guillain-Barre syndrome |
| [Suzuki et al. 2005](https://pubmed.ncbi.nlm.nih.gov/16190970/)^8^ | Case report | 12 years/male | Anti-MP IgM antibody (by PA method) and IgG antibody (by CF method) positive | Fever, cervical lymphadenopathy and erythema multiforme | IgA nephropathy |
| [Termote et al. 2007](https://pubmed.ncbi.nlm.nih.gov/17661054/)^9^ | Case report | 16 years/female | Detection of MP antibodies with seroconversion | Fever | Acute disseminated encephalomyelitis |
| [Greco et al. 2007](https://pubmed.ncbi.nlm.nih.gov/17990593/)^10^ | Case report | 13 years/female | Serum MP antibodies elevated | Leg weakness 2 months after fever, cough, tachypnea and wheezing | Demyelinating polyneuropathy |
| [Kim et al. 2008](https://pubmed.ncbi.nlm.nih.gov/18303215/)^11^ | Case report | 2.5 years/female | MP by serology and PCR | Hematochezia and ecchymotic skin lesion | Acquired hemophilia |
| [Matsumoto et al. 2009](https://pubmed.ncbi.nlm.nih.gov/18809269/)^12^ | Case report | 1 year/female | Increased titer of MP serum antibody  Detection of MP DNA in  initial CSF sample | Pathological findings of characteristic skin lesions, repetitive seizures | Acute disseminated encephalomyelitis |
| [Cassimos et al. 2008](https://pubmed.ncbi.nlm.nih.gov/18850483/)^13^ | Case report | 8 years/male | Identification of specific anti-MP IgM antibodies | Fever and cough | Autoimmune hemolytic anemia |
| [Lacroix et al. 2010](https://pubmed.ncbi.nlm.nih.gov/18922663/)^14^ | Case report | 7 years/female | PCR nasopharyngeal secretion positive for MP | Fever, cough, colicky abdominal pain, and vomiting | Guillain-Barré syndrome |
| [Gursel et al. 2009](https://pubmed.ncbi.nlm.nih.gov/19755923/)^15^ | Case report | 9 years/female | MP antibody titers positive for IgM (31.5 RU/mL) | Dyspnea, cough, and fever | Autoimmune hemolytic anemia, pancytopenia |
| [Graw-Panzer et al. 2009](https://pubmed.ncbi.nlm.nih.gov/19806855/)^16^ | Case report | 13 years/male | MP IgM 5260 U/l | Fever, lethargy, nonproductive cough, nasal congestion, vomiting, diarrhea | Deep vein thrombosis and pulmonary embolism |
| [Akinci et al. 2010](https://pubmed.ncbi.nlm.nih.gov/20718193/)^17^ | Case report | 13 years/female | Positive MP IgM and IgG serology | Ophthalmoplegia, ataxia and areflexia | Miller Fisher syndrome |
| [Nagashima et al. 2010](https://pubmed.ncbi.nlm.nih.gov/20847069/)^18^ | Case report | 9 years/male | High level of MP antibodies 1:10,240 | Cough and fever | Cardiac thrombus |
| [Meyer Sauteur et al. 2011](https://pubmed.ncbi.nlm.nih.gov/21509704/)^19^ | Case report | 3.8 years/female  7 years/male | Acute IgM 1:80; Conv IgM 1:40  Acute IgM 1:320; Conv IgM 1:160 | Respiratory symptoms with fever | Encephalitis |
| [Dhouib et al. 2011](https://pubmed.ncbi.nlm.nih.gov/21780047/)^20^ | Case report | 5 years/male | Cold agglutinin 256 (4°C) and 64 (37°C) units | Jaundice and splenomegaly | Autoimmune hemolytic anemia |
| [Huang et al. 2011](https://pubmed.ncbi.nlm.nih.gov/22057395/)^21^ | Case report | 3.5 years/male | Positive serological tests for MP | Prolonged fever | Kawasaki disease |
| [Nenna et al. 2011](https://pubmed.ncbi.nlm.nih.gov/22185928/)^22^ | Case report | 5 years/female | Chest x-ray and serologic tests showed MP pneumonia | Abdominal pain | Celiac disease |
| [Aleyadhy et al. 2013](https://pubmed.ncbi.nlm.nih.gov/23545616/)^23^ | Case report | 7 years/female | Immunofluorescence test positive for serum MP IgM | Seizure and hypertensive encephalopathy | Guillain-Barré syndrome |
| [Godron et al. 2013](https://pubmed.ncbi.nlm.nih.gov/23800801/)^24^ | Case report | 1 year/male | MP PCR, culture, IgM-ELISA assay all positive | Cough and fever | Hemolytic uremic syndrome |
| [Hanzawa et al. 2013](https://pubmed.ncbi.nlm.nih.gov/24462436/)^25^ | Case report | 3 years/male | MP titer 1: 2560 | Bilateral leg pain, difficulty moving legs spontaneously, fever, seizure | Guillain-Barré syndrome |
| [Venâncio et al. 2014](https://pubmed.ncbi.nlm.nih.gov/25222311/)^26^ | Case report | 9 years/male | MP positive IgM in serum | Limb muscle weakness, fatigue, waddling gait, refusal to walk, changes of mood and self-injurious behavior after upper respiratory symptom and fever | Anti-N-methyl-D-aspartate receptor encephalitis |
| [Miklaszewska et al. 2016](https://pubmed.ncbi.nlm.nih.gov/29693967/)^27^ | Case report | 5 years/female | MP IgM positive | Diarrhea, upper respiratory tract infection | Hemolytic uremic syndrome |
| [Perry et al. 2016](https://pubmed.ncbi.nlm.nih.gov/27079602/)^28^ | Case report | 16 years/female | Elevated MP IgG 4.68 and IgM 2.97 (reference range < 0.90) | Throbbing right eye pain, redness, blurry vision, photophobia, and intermittent headache | Uveitis and perineuritis |
| [Diplomatico et al. 2017](https://pubmed.ncbi.nlm.nih.gov/27921149/)^29^ | Case report | 17 years/female | MP IgM fourfold rise | Symmetrical widespread urticarial eruption | Urticarial vasculitis |
| [Lee et al. 2017](https://pubmed.ncbi.nlm.nih.gov/28336122/)^30^ | Case report | 12 months/female | MP IgM antibody positive (14AU/ml) | Fever, vomiting, seizure | Acute necrotizing encephalopathy |
| [Wandro et al. 2018](https://pubmed.ncbi.nlm.nih.gov/29135841/)^31^ | Case report | 7 months/female | PCR positive for MP | Fever, fatigue, decreased appetite, and pallor | Autoimmune hemolytic anemia |
| [Ting et al. 2018](https://pubmed.ncbi.nlm.nih.gov/29378740/)^32^ | Case report | 5 years/male | MP serology titers > 640 | Fever and cough (2 weeks) | Fuchs syndrome |
| [Matsunaga et al. 2018](https://pubmed.ncbi.nlm.nih.gov/29429558/)^33^ | Case report | 12 years/female | MP particle agglutination titers elevated 1:1280 | Fever and cough | Guillain-Barré syndrome and optic neuritis |
| [Vampertzi et al. 2018](https://pubmed.ncbi.nlm.nih.gov/30262531/)^34^ | Case report | 6 years/female | MP IgM positive | Sudden pallor and pain in both lower limbs | Syringomyelia and Guillain-Barre syndrome |
| [Takahashi et al. 2019](https://pubmed.ncbi.nlm.nih.gov/30270004/)^35^ | Case report | 10 years/male | MP antibody titer 1:240 | Fever, cough, and walking disability | Carotid arteritis and polymyalgia |
| [Bonagiri et al. 2020](https://pubmed.ncbi.nlm.nih.gov/32434879/)^36^ | Case report | 4 years/female | Qualitative serum MP IgM positive | Fever (6 days), mild cough, congestion, weakness | Acute disseminated encephalomyelitis |
| [Müller et al. 2021](https://pubmed.ncbi.nlm.nih.gov/32809980/)^37^ | Case report | 14 years/male | PCR positive  throat swab for MP | Exertional dyspnea, fatigue, painful plaques and papules of face and palms | Kikuchi–Fujimoto disease |
| [Roshan et al. 2020](https://pubmed.ncbi.nlm.nih.gov/32918437/) ^38^ | Case report | 5 years/female | Mycoplasma titer 1:40 (initial); 1:320 (6 days later) | Fever, cough, runny nose, sore throat, reduced oral intake | Autoimmune hemolytic anemia |
| [Sudhakar et al. 2021](https://pubmed.ncbi.nlm.nih.gov/33711641/)^39^ | Case report | 8 years/male | IgM serology for Mycoplasma | Maculopapular rash over trunk and blackish discoloration of right foot | Cold agglutinin disease |

**Supplementary Table 4. Previous cross-sectional and non-controlled observational studies of *M. pneumoniae* infection and autoimmune disease in children**

| **Author/year of publication** | **Type of study** | **Subjects** | **Mean follow- up duration** | **Autoimmune disease (Outcome)** | **Results** |
| --- | --- | --- | --- | --- | --- |
| [Murthy et al. 2002](https://pubmed.ncbi.nlm.nih.gov/12165620/) ^40^ | Observational study | 18 children with acute disseminated encephalomyelitis | N/A | Acute disseminated encephalomyelitis | 3 probable or possible MP diagnoses |
| [Harjacek et al. 2006](https://pubmed.ncbi.nlm.nih.gov/16391883/) ^41^ | Case series | 12 patients with reactive arthritis secondary to acute MP infection | N/A | Juvenile spondyloarthropathies | 4 patients progressed to chronic juvenile spondyloarthropathy |
| [Schessl et al. 2006](https://pubmed.ncbi.nlm.nih.gov/16691408/) ^42^ | Multicenter Study | 95 children with Guillain-Barré syndrome | N/A | Guillain-Barré syndrome | MP frequently involved (7%) |
| [Gorthi et al. 2006](https://pubmed.ncbi.nlm.nih.gov/16836263/) ^43^ | Cross-sectional study | 20 patients with Guillain-Barré syndrome | N/A | Guillain-Barré syndrome | MP infection in 50% of patients vs 25% of household controls vs 15% of hospital controls |
| [Lin et al. 2014](https://pubmed.ncbi.nlm.nih.gov/23143714/) ^44^ | Observational study | 34 children with severe encephalitis | 10.1 ± 4.8 months | Severe acute pediatric encephalitis | 6 with presumed etiologies of MP |
| [Erol et al. 2013](https://pubmed.ncbi.nlm.nih.gov/23849604/)^45^ | Observational study | 15 children with acute disseminated encephalomyelitis | 1.8 ± 1.1 years | Acute disseminated encephalomyelitis | 2 children had serologic evidence of MP |
| [Pillai et al. 2015](https://pubmed.ncbi.nlm.nih.gov/25802349/)^46^ | Observational study | 164 children diagnosed with acute encephalitis | 5.8 years | Infectious and autoantibody-associated encephalitis | MP-associated encephalitis occurred in 7%  * Note that the diagnostic specificity of the method used (mycoplasma IgM) has been questioned. |
| [Meyer Sauteur et al. 2015](https://pubmed.ncbi.nlm.nih.gov/26115201/)^47^ | Case series | 7 children with recent MP infection and severe Guillain-Barré syndrome | N/A | Guillain-Barré syndrome  Bickerstaff brain stem encephalitis  Acute-onset chronic inflammatory demyelinating polyneuropathy | MP infection in children can be followed by severe complicated Guillain-Barré syndrome |
| [Tang et al. 2016](https://pubmed.ncbi.nlm.nih.gov/27609267/) ^48^ | Observational study | 450 patients with KD | 3 years | Kawasaki disease | MP was positive in 62 patients (13.8%) |
| [Iwasa et al. 2018](https://pubmed.ncbi.nlm.nih.gov/29173125/) ^49^ | Observational study | 66 patients with myasthenia gravis | 62.6 months (12–75 months) | Myasthenia gravis | MP influenza likely important trigger for Myasthenia gravis autoimmune activation |
| [Lepri et al. 2019](https://pubmed.ncbi.nlm.nih.gov/31140830/)^50^ | Observational study | 371 immune-mediated encephalopathies | 7 years | Pediatric autoimmune neuropsychiatric disorder associated with *Streptococcus pyogenes* infection (PANDAS)  Pediatric acute-onset neuropsychiatric syndrome (PANS) | Anti-MP antibodies found positive in 11 (42.3%) patients with PANS |
| [Liu et al. 2020](https://pubmed.ncbi.nlm.nih.gov/31948402/)^51^ | Observational study | 43 children with MP-associated thrombosis | N/A | Thrombosis | Severe MP pneumonia associated to MP associated thrombosis |
| [Lan et al. 2020](https://pubmed.ncbi.nlm.nih.gov/32384451/)^52^ | Observational study | 210 patients diagnosed with KD | 24 months | Kawasaki disease | No direct evidence of MP as a trigger of Kawasaki disease |
| [Hussein et al. 2020](https://pubmed.ncbi.nlm.nih.gov/32878734/)^53^ | Prospective study | 44 patients with idiopathic thrombotic thrombocytopenic purpura | N/A | Thrombotic thrombocytopenic purpura | MP was associated with an initial episode in one patient and triggered a relapse in another patient |
| [Poddighe et al. 2021](https://pubmed.ncbi.nlm.nih.gov/34660816/)^54^ | Cross-sectional study | 139 pediatric patients | N/A | Juvenile idiopathic arthritis (JIA) | Seroprevalence rates for MP infection did not different significantly in patients with JIA, rheumatic disorders other than JIA, and endocrinological and non-inflammatory conditions |

**Supplementary Table 5. Previous Case-Control studies of *M. pneumoniae* infection and autoimmune diseases in children**

| **Author/year of publication** | **Type of study** | **Subjects** | **Mean follow- up duration** | **Autoimmune disease (Outcome)** | **Results** |
| --- | --- | --- | --- | --- | --- |
| [Sharma et al. 2011](https://pubmed.ncbi.nlm.nih.gov/21727645/)^55^ | Cross-sectional study | 57 pediatric GBS patients, 42 neurological controls and 35 non-neurological controls | N/A | Guillain-Barré syndrome | Significant difference in levels of MP IgG antibodies between GBS patients and neurological controls (P < 0.05). |
| [Tang et al. 2016](https://pubmed.ncbi.nlm.nih.gov/26884440/)^56^ | Case-control study | 1016 Kawasaki disease patients, 12 of whom had MP infection | 9 years | Kawasaki Disease | MP group vs non-MP group in patients with KD showed no significant difference for coronary aneurysm incidence |
| [Meyer Sauteur et al. 2016](https://pubmed.ncbi.nlm.nih.gov/27490360/)^57^ | Case-control study | 189 adults and 24 children with GBS vs control cohorts (n=479) | N/A | Guillain-Barré syndrome | MP infection is associated with GBS, more frequently in children |
| [Meyer Sauteur et al. 2018](https://pubmed.ncbi.nlm.nih.gov/29301655/)^58^ | Case-control study | 13 MP-GBS patients along with 25 MP-negative GBS controls and 7 non-GBS controls | N/A | Guillain-Barré syndrome | Intrathecal anti-MP antibody production and CNS symptoms in GBS patients indicate that CNS infection may occur before or during GBS development |
| [Ding et al. 2021](https://pubmed.ncbi.nlm.nih.gov/34160770/)^59^ | Retrospective observational study | 4742 patients, who underwent MP and virus detection classified into groups of Kawasaki disease (n=98) and non- Kawasaki disease (n=4644) | N/A | Kawasaki disease | MP positive rate  ≤3 years, significantly higher;  >3 years not statistically significant |

**Supplementary Table 6. Terms used for searching PubMed**

| 1 | "Mycoplasma"[Mesh] | 17,645 |
| --- | --- | --- |
| 2 | mycoplasma* | 29,278 |
| 3 | ("Mycoplasma"[Mesh]) OR (mycoplasma*) | 29,278 |
| 4 | MESH terms for Autoimmune disease (See Supplementary Table 2) |  |
| 5 | Mycoplasma AND Autoimmune disease |  |

**Supplementary Table 7.  Risk of autoimmune disease in the exposed cohort (****patients admitted with *M. pneumoniae* infection) based on different indicators of infection**

|  | No. of autoimmune disease cases/No. of accumulated person-years ⋅ 10,000 (incidence rate/10,000 person-years) | | Absolute rate difference/10,000 person-years  (95% CI) ^a^ | Hazard ratio (95% CI)^b^ | *P* value^c^ |
| --- | --- | --- | --- | --- | --- |
| *M. pneumoniae* infection care indicators | Exposed individuals | Matched unexposed individuals |  |  |  |
| Use of antibiotics ^d,e^ |  |  |  |  |  |
| No | 3 / 0.04 (82.3) | 6,604/148 (44.7) | - | 1.783 (0.575–5.529) | 0.48 |
| Yes | 3,292/49 (66.5) | 19,541/352 (55.5) | 11.00 (8.60–13.40) | 1.183 (1.139-1.228) |  |
| Oxygen therapy ^d^ |  |  |  |  |  |
| No | 3,156/48 (66.4) | 25,925/496 (52.3) | 14.05 (11.65–16.46) | 1.259 (1.213-1.307) | 0.91 |
| Yes | 139/2 (71.0) | 4031/4 (55.0) | 15.91 (2.07–29.77) | 1.314 (1.055-1.637) |  |
| Frequency of outpatient visits within a month after index date^f^ |  |  |  |  |  |
| No | 27/0 (56.2) | 4,412/108 (40.8) | - | 1.057 (0.689-1.624) | 0.52 |
| Yes | 3,268/49 (66.6) | 21,733/392 (55.5) | 11.15 (8.75–13.55) | 1.193 (1.149-1.239) |  |

^a^ Results were not calculated if the number of cases was fewer than 10.

^b^ Cox models were stratified by matching identifiers (birth year and sex) and adjusted for birth residence (Seoul/metropolitan, city, or rural area), household income (low, middle, or high), perinatal history (disorders related to fetal growth and development, birth injury, congenital malformations), and prescription duration of medications (macrolide antibiotics and systemic steroids). The first year of follow-up was excluded from all analyses.

^c^ *P* value was from an interaction test, by incorporating an interaction term to the Cox model.

^d^ Prescription from 1 month before to 1 month after the index date.

^e^ The median prescription duration was 11 days.

^f^ The median outpatient visit event was 3 days.

**Supplementary Table 8. Data used for the meta-analysis (Figure 3) of the association between *M. pneumoniae* infection and autoimmune diseases from the Common Data Model (CDM)**

|  | Study period | Exposed cohort, N /Matched unexposed cohort, N (total cohort, N) |  | Follow-up time, y | |  | Any autoimmune disease | |  | Incidence rate/1,000 person-years | |  |  |
| --- | --- | --- | --- | --- | --- | --- | --- | --- | --- | --- | --- | --- | --- |
|  |  |  |  | Exposed | Unexposed |  | Exposed | Unexposed |  | Exposed | Unexposed |  | HR (95% CI)^a^ |
| MJ | 2003-2020 | 1,678/10,511 (880,392) |  | 8,349 | 51,939 |  | 113 | 607 |  | 13.5 | 11.7 |  | 1.19 (0.94–1.50) |
| KH | 2006-2017 | 877/5,524 (822,183) |  | 3,406 | 21,380 |  | 22 | 133 |  | 6.5 | 6.2 |  | 0.95 (0.53–1.70) |
| CHA | 2006-2019 | 2,325/19,158 (2,363,386) |  | 7,705 | 62,408 |  | 62 | 401 |  | 9.1 | 6.4 |  | 1.42 (1.07–1.89) |
| PS | 2011-2018 | 359/2,447 (1,753,001) |  | 1,183 | 7,669 |  | 7 | 36 |  | 5.9 | 4.7 |  | 1.58 (0.63–3.99) |

Abbreviations: CHA, Bundang CHA Medical Center; CI, confidence interval; KH, Kyung Hee University Hospital at Gangdong; MJ, Myongji Hospital; N, number; PS, Pusan National University Hospital; y, year.

^a^ Cox models were stratified by matching identifiers (birth year and sex) and adjusting for birth residence (Seoul/metropolitan, city, or rural area), household income (low, middle, or high), perinatal history (disorders related to fetal growth and development, birth injury, congenital malformations), and duration of medication use (macrolide antibiotics and systemic steroids). The first year of follow-up was excluded from all analyses.

**Supplementary Table 9. Characteristics of participants included in the Common Data Model (CDM) analysis at each hospital**

|  | **Unmatched cohort** |  |  | **Matched cohort** |  |  |
| --- | --- | --- | --- | --- | --- | --- |
| **Characteristic, %** | **Exposed** | **Unexposed** | **SD** | **Exposed** | **Unexposed** | **SD** |
| **MJ** |  |  |  |  |  |  |
| Age group (yr) |  |  |  |  |  |  |
| 1–4 | 47.3 | 13.2 | 0.80 | 48.7 | 52.2 | 0.07 |
| 5–9 | 42.2 | 3.9 | 1.02 | 41.5 | 37.9 | 0.08 |
| 10–14 | 8.6 | 2.8 | 0.25 | 8.0 | 8.3 | 0.01 |
| 15–19 | 2.0 | 3.9 | 0.11 | 1.7 | 1.6 | 0.01 |
| Sex |  |  |  |  |  |  |
| Female | 51.3 | 47.9 | 0.07 | 53.5 | 51.7 | 0.04 |
| Race |  |  |  |  |  |  |
| Korean | 99.8 | 98.4 | 0.15 | 99.8 | 99.5 | 0.06 |
| **KH** |  |  |  |  |  |  |
| Age group (yr) |  |  |  |  |  |  |
| 1–4 | 46.3 | 11.4 | 0.84 | 52.3 | 54.0 | 0.03 |
| 5–9 | 45.0 | 3.0 | 1.13 | 40.5 | 39.8 | 0.01 |
| 10–14 | 7.8 | 1.7 | 0.29 | 6.3 | 5.4 | 0.04 |
| 15–19 | 0.9 | 2.6 | 0.13 | 0.9 | 0.7 | 0.02 |
| Sex |  |  |  |  |  |  |
| Female | 51.9 | 54.2 | 0.05 | 51.5 | 51.4 | 0.00 |
| Race |  |  |  |  |  |  |
| Korean | 99.7 | 98.1 | 0.15 | 99.8 | 99.4 | 0.06 |
| **CHA** |  |  |  |  |  |  |
| Age group (yr) |  |  |  |  |  |  |
| 1–4 | 58.5 | 20.0 | 0.86 | 63.2 | 64 | 0.03 |
| 5–9 | 32.8 | 4.6 | 0.78 | 30.0 | 28 | 0.03 |
| 10–14 | 7.4 | 1.9 | 0.26 | 5.8 | 6 | 0.01 |
| 15–19 | 1.3 | 2.5 | 0.09 | 1.1 | 1 | 0.00 |
| Sex |  |  |  |  |  |  |
| Female | 51.7 | 57.3 | 0.11 | 51.2 | 50 | 0.02 |
| Race |  |  |  |  |  |  |
| Korean | 99.1 | 98.2 | 0.08 | 99.0 | 99 | 0.03 |
| **PS** |  |  |  |  |  |  |
| Age group (yr) |  |  |  |  |  |  |
| 1–4 | 51.8 | 5.6 | 1.19 | 56.8 | 56.2 | 0.01 |
| 5–9 | 30.0 | 1.8 | 0.84 | 25.6 | 26.1 | 0.01 |
| 10–14 | 11.4 | 1.5 | 0.41 | 10.9 | 10.3 | 0.02 |
| 15–19 | 6.7 | 2.3 | 0.21 | 6.7 | 7.3 | 0.02 |
| Sex |  |  |  |  |  |  |
| Female | 44.6 | 48.2 | 0.07 | 40.4 | 43.3 | 0.06 |
| Race |  |  |  |  |  |  |
| Korean | 99.6 | 98.9 | 0.08 | 100.0 | 98.7 | 0.16 |

**Abbreviations:** CHA, Bundang CHA medical center; CI, confidence interval; KH, Kyung Hee University Hospital at Gangdong; MJ, Myongji Hospital; SD, standard deviation.

**Supplementary Figure 1. Incidence of *M. pneumoniae* infections in children of different ages during the study period**

**
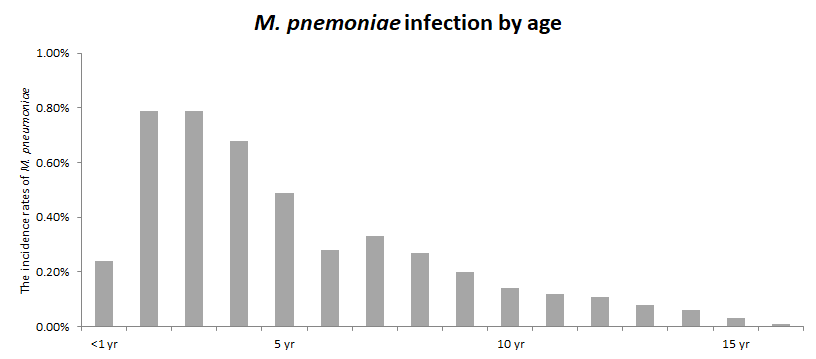
**

**Supplementary Figure 2. Trends in *M. pneumoniae* infection-related hospitalization from 2002 to 2016**

**Supplementary Figure 3. Cumulative incidence rates of autoimmune diseases for participants in the exposed group (history of hospitalization due to *M. pneumoniae* infection) and the matched unexposed group**


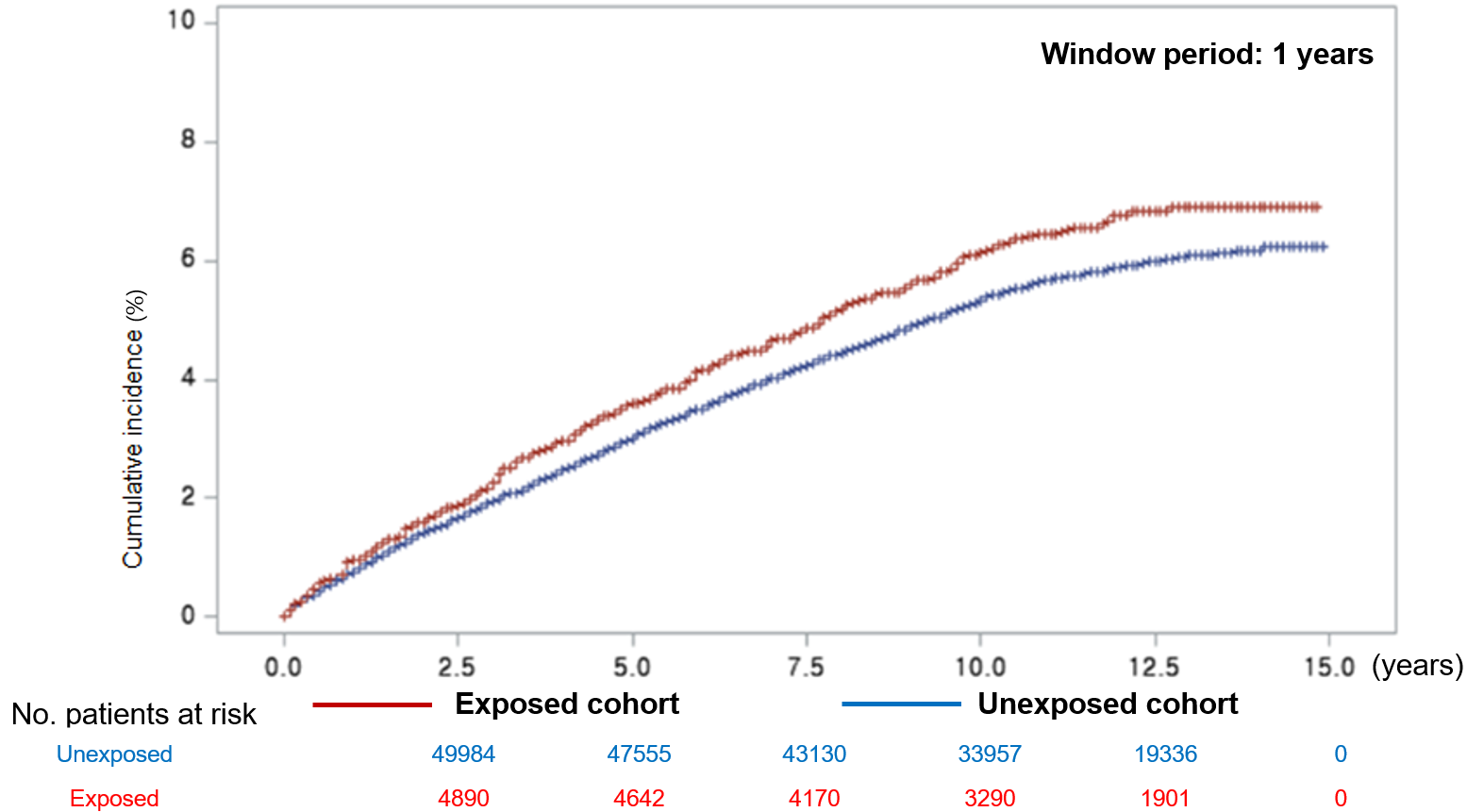


Cumulative incidence (%) of autoimmune diseases in the exposed and unexposed cohort. The window period was 1 years and 10% of the participants with more than a window period of follow-up after hospitalization due to *M. pneumoniae* infection were randomly selected for this analysis. The discrepancies in the numbers of the selected 10% of the exposed and unexposed groups are due to the window period and cases in which participants transitioned from the non-exposed group to the exposed group, resulting in necessary adjustments.

**REFERENCE**

1. Sakoulas G. Brainstem and striatal encephalitis complicating Mycoplasma pneumoniae pneumonia: possible benefit of intravenous immunoglobulin. Pediatr Infect Dis J. 2001;20(5):543-5.

2. Chryssanthopoulos C, Eboriadou M, Monti K, Soubassi V, Sava K. Fatal disseminated intravascular coagulation caused by Mycoplasma pneumoniae. Pediatr Infect Dis J. 2001;20(6):634-5.

3. Wang JN, Wang SM, Liu CC, Wu JM. Mycoplasma pneumoniae infection associated with Kawasaki disease. Acta Paediatr. 2001;90(5):594-5.

4. Ashtekar CS, Jaspan T, Thomas D, Weston V, Gayatri NA, Whitehouse WP. Acute bilateral thalamic necrosis in a child with Mycoplasma pneumoniae. Dev Med Child Neurol. 2003;45(9):634-7.

5. Hsueh KC, Chou IC, Hsu CH, Kuo HT, Tsai FJ, Tsai CH. Miller Fisher syndrome possibly related to mycoplasma pneumoniae infection: report of one case. Acta Paediatr Taiwan. 2004;45(3):168-70.

6. Aydin A, Atasever S, Cakmakci H. Acute disseminated encephalomyelitis presenting with bilateral transient amaurosis. Pediatr Neurol. 2005;32(1):60-3.

7. Arakawa H, Yuhara Y, Todokoro M, Kato M, Mochizuki H, Tokuyama K, et al. Immunoadsorption therapy for a child with Guillain-Barre syndrome subsequent to Mycoplasma infection: a case study. Brain Dev. 2005;27(6):431-3.

8. Suzuki K, Hirano K, Onodera N, Takahashi T, Tanaka H. Acute IgA nephropathy associated with mycoplasma pneumoniae infection. Pediatr Int. 2005;47(5):583-5.

9. Termote B, Demaerel P, Wilms G, Dubois B. Encephalitis following Mycoplasma pneumonia (2007: 6b). Acute disseminated encephalomyelitis. Eur Radiol. 2007;17(9):2436-8.

10. Greco F, Salvo V, Sorge A, Perrini S, Garozzo R, Sorge G. Co-infection with Mycoplasma pneumoniae and cytomegalovirus resulting in an acute demyelinating polyneuropathy in a pediatric patient. Turk J Pediatr. 2007;49(3):331-3.

11. Kim MS, Kilgore PE, Kang JS, Kim SY, Lee DY, Kim JS, et al. Transient acquired hemophilia associated with Mycoplasma pneumoniae pneumonia. J Korean Med Sci. 2008;23(1):138-41.

12. Matsumoto N, Takahashi S, Toriumi N, Sarashina T, Makita Y, Tachibana Y, et al. Acute disseminated encephalomyelitis in an infant with incontinentia pigmenti. Brain Dev. 2009;31(8):625-8.

13. Cassimos D, Bezirgiannidou Z, Pantelidou D, Christoforidis A, Chatzimichael A, Maritinis G. Warm autoimmune hemolytic anemia following recurrent mycoplasma pneumonia infections in a child with Down syndrome. Pediatr Hematol Oncol. 2008;25(7):693-8.

14. Lacroix LE, Galetto A, Haenggeli CA, Gervaix A. Delayed recognition of Guillain-Barre syndrome in a child: a misleading respiratory distress. J Emerg Med. 2010;38(5):e59-61.

15. Gursel O, Altun D, Atay AA, Bedir O, Kurekci AE. Mycoplasma pneumoniae infection associated with pancytopenia: a case report. J Pediatr Hematol Oncol. 2009;31(10):760-2.

16. Graw-Panzer KD, Verma S, Rao S, Miller ST, Lee H. Venous thrombosis and pulmonary embolism in a child with pneumonia due to Mycoplasma pneumoniae. J Natl Med Assoc. 2009;101(9):956-8.

17. Akinci G, Oztura I, Hiz-Kurul S. Anti-GQ1b-negative Miller Fisher syndrome presented with one-sided horizontal gaze palsy. Turk J Pediatr. 2010;52(3):317-20.

18. Nagashima M, Higaki T, Satoh H, Nakano T. Cardiac thrombus associated with Mycoplasma pneumoniae infection. Interact Cardiovasc Thorac Surg. 2010;11(6):849-51.

19. Meyer Sauteur PM, Streuli JC, Iff T, Goetschel P. Mycoplasma pneumoniae-associated encephalitis in childhood--nervous system disorder during or after a respiratory tract infection. Klin Padiatr. 2011;223(4):209-13.

20. Dhouib N, Guedhami H, Mellouli F, Ben Khaled M, Kaabi H, Belhassen E, et al. Mycoplasma pneumoniae associated with severe autoimmune hemolytic anemia in a child with homozygous beta-thalassemia. Tunis Med. 2011;89(7):652-3.

21. Huang FL, Chang TK, Jan SL, Tsai CR, Wang LC, Lai MC, et al. Co-morbidity of Kawasaki disease. Indian J Pediatr. 2012;79(6):815-7.

22. Nenna R, D'Eufemia P, Celli M, Mennini M, Petrarca L, Zambrano A, et al. Celiac disease and lamellar ichthyosis. Case study analysis and review of the literature. Acta Dermatovenerol Croat. 2011;19(4):268-70.

23. Aleyadhy AA, Hassan GM. Hypertensive encephalopathy as the initial manifestation of Guillain-Barre syndrome in a 7-year-old girl. Neurosciences (Riyadh). 2013;18(2):163-5.

24. Godron A, Pereyre S, Monet C, Llanas B, Harambat J. Hemolytic uremic syndrome complicating Mycoplasma pneumoniae infection. Pediatr Nephrol. 2013;28(10):2057-60.

25. Hanzawa F, Fuchigami T, Ishii W, Nakajima S, Kawamura Y, Endo A, et al. A 3-year-old boy with Guillain-Barre syndrome and encephalitis associated with Mycoplasma pneumoniae infection. J Infect Chemother. 2014;20(2):134-8.

26. Venancio P, Brito MJ, Pereira G, Vieira JP. Anti-N-methyl-D-aspartate receptor encephalitis with positive serum antithyroid antibodies, IgM antibodies against mycoplasma pneumoniae and human herpesvirus 7 PCR in the CSF. Pediatr Infect Dis J. 2014;33(8):882-3.

27. Miklaszewska M, Zachwieja K, Drozdz D, Pallinger E, Takacs B, Szilagyi A, et al. Hemolytic uremic syndrome with Mycoplasma pneumoniae infection and membrane cofactor protein mutation - case report. Przegl Lek. 2016;73(11):862-4.

28. Perry JT, Chen WS. Acute Mycoplasma pneumoniae infection presenting with unilateral anterior uveitis and perineuritis. J AAPOS. 2016;20(2):178-80.

29. Diplomatico M, Gicchino MF, Ametrano O, Marzuillo P, Olivieri AN. A case of urticarial vasculitis in a female patient with lupus: Mycoplasma pneumoniae infection or lupus reactivation? Rheumatol Int. 2017;37(5):837-40.

30. Lee YJ, Hwang SK, Lee SM, Kwon S. Familial acute necrotizing encephalopathy with RANBP2 mutation: The first report in Northeast Asia. Brain Dev. 2017;39(7):625-8.

31. Wandro C, Dolatshahi L, Blackall D. Severe Warm Autoimmune Hemolytic Anemia in a 7-Month-Old Infant Associated With a Mycoplasma Pneumoniae Infection. J Pediatr Hematol Oncol. 2018;40(7):e439-e41.

32. Ting CY, Shahdadpuri R. Fuchs syndrome: a case of fever, mucositis and conjunctivitis. BMJ Case Rep. 2018;2018.

33. Matsunaga M, Kodama Y, Maruyama S, Miyazono A, Seki S, Tanabe T, et al. Guillain-Barre syndrome and optic neuritis after Mycoplasma pneumoniae infection. Brain Dev. 2018;40(5):439-42.

34. Vampertzi O, Dalpa E, Vavilis T, Tramma D. Comorbid presentation of syringomyelia and Guillain-Barre syndrome, attributed to mycoplasma, in a 6-year-old female patient. BMJ Case Rep. 2018;2018.

35. Takahashi I, Ishihara M, Oishi T, Yamamoto M, Narita M, Fujieda M. Common carotid arteritis and polymyalgia with Mycoplasma pneumoniae infection. J Infect Chemother. 2019;25(4):281-4.

36. Bonagiri P, Park D, Ingebritsen J, Christie LJ. Seropositive anti-MOG antibody-associated acute disseminated encephalomyelitis (ADEM): a sequelae of Mycoplasma pneumoniae infection. BMJ Case Rep. 2020;13(5).

37. Muller CSL, Vogt T, Becker SL. Kikuchi-Fujimoto Disease Triggered by Systemic Lupus Erythematosus and Mycoplasma pneumoniae Infection-A Report of a Case and a Review of the Literature. Am J Dermatopathol. 2021;43(3):202-8.

38. Roshan S, Tan SW. A case report of severe mycoplasma pneumonia with autoimmune haemolytic anaemia. Med J Malaysia. 2020;75(5):600-2.

39. Sudhakar M, Mohandoss V, Chaudhary H, Ahluwalia J, Bhattarai D, Jindal AK. Multifocal thrombosis with peripheral gangrene in a young boy: Mycoplasma infection triggered cold agglutinin disease. Immunobiology. 2021;226(3):152075.

40. Murthy SN, Faden HS, Cohen ME, Bakshi R. Acute disseminated encephalomyelitis in children. Pediatrics. 2002;110(2 Pt 1):e21.

41. Harjacek M, Ostojic J, Djakovic Rode O. Juvenile spondyloarthropathies associated with Mycoplasma pneumoniae infection. Clin Rheumatol. 2006;25(4):470-5.

42. Schessl J, Luther B, Kirschner J, Mauff G, Korinthenberg R. Infections and vaccinations preceding childhood Guillain-Barre syndrome: a prospective study. Eur J Pediatr. 2006;165(9):605-12.

43. Gorthi SP, Kapoor L, Chaudhry R, Sharma N, Perez-Perez GI, Panigrahi P, et al. Guillain-Barre syndrome: association with Campylobacter jejuni and Mycoplasma pneumoniae infections in India. Natl Med J India. 2006;19(3):137-9.

44. Lin JJ, Lin KL, Chiu CH, Hsia SH, Wang HS, Chou IJ, et al. Antineuronal antibodies and infectious pathogens in severe acute pediatric encephalitis. J Child Neurol. 2014;29(1):11-6.

45. Erol I, Ozkale Y, Alkan O, Alehan F. Acute disseminated encephalomyelitis in children and adolescents: a single center experience. Pediatr Neurol. 2013;49(4):266-73.

46. Pillai SC, Hacohen Y, Tantsis E, Prelog K, Merheb V, Kesson A, et al. Infectious and autoantibody-associated encephalitis: clinical features and long-term outcome. Pediatrics. 2015;135(4):e974-84.

47. Meyer Sauteur PM, Roodbol J, Hackenberg A, de Wit MC, Vink C, Berger C, et al. Severe childhood Guillain-Barre syndrome associated with Mycoplasma pneumoniae infection: a case series. J Peripher Nerv Syst. 2015;20(2):72-8.

48. Tang Y, Yan W, Sun L, Huang J, Qian W, Hou M, et al. Kawasaki disease associated with Mycoplasma pneumoniae. Ital J Pediatr. 2016;42(1):83.

49. Iwasa K, Yoshikawa H, Hamaguchi T, Sakai K, Shinohara-Noguchi M, Samuraki M, et al. Time-series analysis: variation of anti-acetylcholine receptor antibody titer in myasthenia gravis is related to incidence of Mycoplasma pneumoniae and influenza virus infections. Neurol Res. 2018;40(2):102-9.

50. Lepri G, Rigante D, Bellando Randone S, Meini A, Ferrari A, Tarantino G, et al. Clinical-Serological Characterization and Treatment Outcome of a Large Cohort of Italian Children with Pediatric Autoimmune Neuropsychiatric Disorder Associated with Streptococcal Infection and Pediatric Acute Neuropsychiatric Syndrome. J Child Adolesc Psychopharmacol. 2019;29(8):608-14.

51. Liu J, He R, Wu R, Wang B, Xu H, Zhang Y, et al. Mycoplasma pneumoniae pneumonia associated thrombosis at Beijing Children's hospital. BMC Infect Dis. 2020;20(1):51.

52. Lan Y, Li S, Yang D, Zhou J, Wang Y, Wang J, et al. Clinical characteristics of Kawasaki disease complicated with Mycoplasma pneumoniae pneumonia: A retrospective study. Medicine (Baltimore). 2020;99(19):e19987.

53. Hussein EA. Idiopathic TTP in the Middle East: Epidemiology and clinical outcomes in infection associated episodes. Transfus Apher Sci. 2020;59(6):102916.

54. Poddighe D, Abdukhakimova D, Dossybayeva K, Mukusheva Z, Assylbekova M, Rakhimzhanova M, et al. Mycoplasma pneumoniae Seroprevalence and Total IgE Levels in Patients with Juvenile Idiopathic Arthritis. J Immunol Res. 2021;2021:6596596.

55. Sharma MB, Chaudhry R, Tabassum I, Ahmed NH, Sahu JK, Dhawan B, et al. The presence of Mycoplasma pneumoniae infection and GM1 ganglioside antibodies in Guillain-Barre syndrome. J Infect Dev Ctries. 2011;5(6):459-64.

56. Tang Y, Gao X, Shen J, Sun L, Yan W. Epidemiological and Clinical Characteristics of Kawasaki Disease and Factors Associated with Coronary Artery Abnormalities in East China: Nine Years Experience. J Trop Pediatr. 2016;62(2):86-93.

57. Meyer Sauteur PM, Huizinga R, Tio-Gillen AP, Roodbol J, Hoogenboezem T, Jacobs E, et al. Mycoplasma pneumoniae triggering the Guillain-Barre syndrome: A case-control study. Ann Neurol. 2016;80(4):566-80.

58. Meyer Sauteur PM, Huizinga R, Tio-Gillen AP, Drenthen J, Unger WWJ, Jacobs E, et al. Intrathecal antibody responses to GalC in Guillain-Barre syndrome triggered by Mycoplasma pneumoniae. J Neuroimmunol. 2018;314:13-6.

59. Ding YY, Ren Y, Qin J, Qian GH, Tang YJ, Chen Y, et al. Clinical characteristics of Kawasaki disease and concurrent pathogens during isolation in COVID-19 pandemic. World J Pediatr. 2021;17(3):263-71.
